# Exploring the Effects of Palm Tocotrienol-Rich Fraction in Diabetic Peripheral Neuropathy Rat’s Model: An Untargeted Metabolomic Profiling and Correlation Study

**DOI:** 10.3390/ijms262311247

**Published:** 2025-11-21

**Authors:** Noradliyanti Rusli, Jen Kit Tan, Suzana Makpol, Isma Liza Mohd Isa, Nur Haleeda Hakimi, Nazirah Ab Rani, Rabani Remli

**Affiliations:** 1 Department of Medicine, Faculty of Medicine, Universiti Kebangsaan Malaysia, Kuala Lumpur 56000, Malaysia; p116652@siswa.ukm.edu.my; 2 Department of Biochemistry, Faculty of Medicine, Universiti Kebangsaan Malaysia, Kuala Lumpur 56000, Malaysia; jenkittan@ukm.edu.my (J.K.T.); suzanamakpol@hctm.ukm.edu.my (S.M.); haleeda@ukm.edu.my (N.H.H.); nazirahrani@ukm.edu.my (N.A.R.); 3 Ageing and Degenerative Diseases Research Group, Universiti Kebangsaan Malaysia, Bangi 43600, Malaysia; 4 Department of Anatomy, Faculty of Medicine, Universiti Kebangsaan Malaysia, Kuala Lumpur 56000, Malaysia; ismaliza.mohdisa@ukm.edu.my; 5 CÚRAM Research Ireland Centre for Medical Devices, School of Medicine, University of Galway, H91 TK33 Galway, Ireland

**Keywords:** diabetic peripheral neuropathy, metabolomic analysis, palm tocotrienol-rich fraction, antioxidants, rat model, correlation study

## Abstract

Persistent and chronic hyperglycaemia in Type II diabetic mellitus (DM) is known to cause oxidative stress, which exacerbates underlying metabolic disorders, contributing to the progression of complications such as diabetic peripheral neuropathy (DPN). Palm tocotrienol-rich fraction (TRF) is renowned for its potent antioxidative and neuroprotective properties and might have the potential to halt or mitigate the severity of DPN. This study aimed to investigate the effects of palm TRF on diabetic rats with peripheral neuropathy and to identify the correlation between plasma metabolomic alterations and DPN parameters. Male Sprague Dawley (SD) rats were randomly divided into normal control and DM groups in which Type II DM was induced using a high-fat diet and a low-dose streptozotocin (STZ) (35 mg/kg). Successful diabetic rats were randomly divided and received daily oral treatments of palm olein (vehicle), metformin (70 mg/kg), TRF (60 mg/kg), or a combination of TRF and metformin for 12 weeks. Behavioural parameters, serum biomarkers, and plasma metabolomic profiling were assessed at 0 (baseline) and 12 weeks of intervention. From the behavioural parameters, improvement in the symptoms of thermal hyperalgesia and mechanical allodynia was seen with TRF interventions, either alone or in combination with metformin. A significant reduction in the neurofilament light (NEFL) chain, accompanied by a notable increase in nerve growth factor (NGF) levels in the serum of treatment groups, was also observed. From the plasma samples, findings reveal that TRF increases metabolites related to neurotransmitter pathways (acetylcholine, choline, phenylalanine, tryptophan) and decreases inflammatory metabolites (kynurenine, prostaglandin) compared to untreated diabetic rats. These metabolites, except for prostaglandin, showed positive correlations with pain sensitivity. In contrast, prostaglandin showed opposite correlations with pain and nerve damage markers, suggesting its potential role in inflammation and axonal injury.

## 1. Introduction

Diabetic peripheral neuropathy (DPN) is a prevalent and debilitating complication of diabetes mellitus (DM), especially Type II DM, which affects a significant proportion of individuals worldwide. It is estimated that the number of individuals affected by Type II DM will increase to 628.6 million by 2045, with the prevalence reaching 9.9% from 6.38% of the world’s population [1,2]. The prevalence of DPN is estimated to be as high as 50%, with higher rates in Type II DM compared to Type I DM [3]. Regional variations in the prevalence have also been observed, with Iran and Europe showing higher prevalence worldwide [4,5]. DPN manifests due to chronic hyperglycaemia and metabolic dysfunction, leading to progressive nerve damage predominantly in the peripheral nervous system [6].

The clinical manifestations of DPN include a spectrum of symptoms such as numbness, tingling, burning pain, and muscle weakness, primarily in the lower extremities [7]. It tends to develop slowly and insidiously, often starting in the feet and spreading upwards in a “stocking and glove” distribution [8]. The symptoms may also include painful manifestations, including hyperalgesia, allodynia, and spontaneous burning or tingling sensations [9]. Due to sensory loss, patients are at a higher risk of foot ulcers, infections, and even amputation of a limb [10]. These conditions significantly impair quality of life and pose a substantial burden on healthcare systems. Due to its impact on patients’ quality of life and the associated healthcare costs, DPN has become an emerging concern as these disease complications and manifestations should be considered significant public health issues [5].

The pathophysiology of DPN is multifaceted, involving a combination of metabolic, vascular, and inflammatory mechanisms. Chronic hyperglycaemia, hyperlipidaemia, oxidative stress, mitochondrial dysfunction, and the accumulation of advanced glycation end products (AGEs), all contribute to axon degeneration, demyelination, and, ultimately, neuronal damage [11,12]. Activation of various pathways, such as the polyol, hexosamine, and protein kinase C pathways, worsen the progression of DPN in Type II DM patients [13,14]. Moreover, impaired blood flow and endothelial dysfunction, due to the resulting free radicals and the above-mentioned pathways, exacerbate the condition by compromising the nutrient and oxygen supply to the peripheral nerves [15].

Current therapeutic options for DPN are limited and primarily focus on symptomatic relief rather than addressing the underlying causes. As a result, there is a growing interest in identifying novel therapeutic agents that can target the fundamental mechanisms of DPN. One such promising candidate is palm tocotrienol-rich fraction (TRF), a natural form of vitamin E derived from palm oil, which consists of a natural mixture of tocotrienols (α-, β-, γ-, δ-) and tocopherols. About 75% of the vitamin E from palm oil consists of tocotrienols, which exhibit potent antioxidant, anti-inflammatory, cardioprotective, and neuroprotective properties [16,17,18,19,20]. Studies have found that tocotrienol may possess 40–60 times greater antioxidant properties than tocopherol, and it also exhibits more potent anti-inflammatory activity [17]. Although α-tocotrienol is recognised as the most potent neuroprotective isomer, the combination present in TRF enhances overall efficacy, reflects natural dietary intake, and is practical for translational application. In addition, recent studies have highlighted the potential of TRF in mitigating oxidative stress and inflammation, both of which play pivotal roles in the pathogenesis of DPN [19,20,21]. Studies have also shown that TRF modulates various biochemical pathways, including the suppression of NF-κB, pro-inflammatory cytokines, and prostaglandin E2 in macrophages [22]. Recently, these pro-inflammatory cytokines have been shown to play a role in the pathogenesis of DPN in SD rats where the activation of NF-κB drives the release of TNF-α and interleukins. In the study, they find that Mer tyrosine kinase (MerTK), a receptor that normally restrains NF-κB signalling, worsened neuropathic damage in diabetic rats, underscoring the importance of targeting inflammatory pathways in DPN [23]. However, although the antioxidant and anti-inflammatory properties of palm TRF have been reported, its specific role in alleviating the symptoms of DPN has not been extensively elucidated. Moreover, the molecular pathways through which TRF exerts these effects in DPN remain unclear and require further investigation. Recent clinical studies have explored the therapeutic potential of tocotrienol-rich vitamin E. A phase II double-blind randomised controlled trial demonstrated that tocotrienol-rich vitamin E (Tocovid) improved nerve conduction velocity in patients with type 2 diabetes mellitus, suggesting beneficial effects on diabetic neuropathy [24]. Similarly, vitamin E supplementation has been investigated in a phase III clinical trial for the prevention of chemotherapy-induced peripheral neuropathy, providing evidence of its neuroprotective properties [25]. These findings underscore the clinical relevance of vitamin E in neuropathic conditions. However, despite these promising outcomes, the precise metabolic mechanisms underlying its effects remain poorly understood. To address this gap, our study employed a type 2 diabetes animal model combined with untargeted metabolomic profiling to investigate the metabolic pathways and potential mediators through which palm TRF may exert protective effects in DPN. This approach allows for a more comprehensive understanding of TRF’s mechanisms, providing novel insights beyond the behavioural and biochemical outcomes previously reported. Given the complexity of DPN and the need for effective therapeutic interventions, there is a compelling need to investigate the therapeutic potential of palm TRF in this context. In this study, metformin was included as a reference treatment because it represents the first-line therapy for Type II DM. While it is not primarily indicated for neuropathy, accumulating evidence suggests that metformin may exert neuroprotective effects and alleviate DPN’s symptoms [26]. Previous experimental studies have shown that metformin may alleviate neuropathic symptoms, likely through its glycaemic control, antioxidant, and anti-inflammatory effects that indirectly support peripheral nerve function [27].

Recently, omics studies that investigate various biological molecules and their roles in systems biology have attracted the interest of researchers, with metabolomic studies focusing on identifying changes in metabolite levels during a body’s complex process [28]. These recent metabolomic studies have also provided valuable insights into the pathogenesis and changes occurring in DPN. Research has revealed significant disturbances in multiple metabolic pathways, including the tricarboxylic acid cycle (TCA), amino acid metabolism, and lipid metabolism, which are associated with nervous system injuries in DPN [6]. A targeted metabolomic approach identified aberrant energy status in DPN patients and animal models, highlighting excessive glycolysis and impaired ATP generation [29]. Longitudinal studies in animal models have shown reduced levels of TCA cycle intermediates, including citric acid, ketoglutaric acid, and succinic acid, in the sciatic nerves of diabetic mice. Additionally, increased levels of branched-chain amino acids and other amino acids have been observed in the serum of diabetic animals [30]. However, no study has been conducted to identify the effect of palm TRF on the metabolomic profile of a DPN animal model, especially regarding the metabolites affected in DPN.

Thus, this study aimed to explore the effects of palm TRF on DPN through untargeted metabolomic profiling of the plasma samples from a DPN animal model. The effect of palm TRF on behavioural and blood biomarkers of DPN and their correlation with the plasma metabolomic profile will also be elucidated in this study. By employing advanced metabolomic techniques, this study seeks to elucidate the metabolic alterations modulated by palm TRF supplementation and uncover potential biomarkers associated with its therapeutic efficacy. The ultimate goal is to provide a comprehensive understanding of the mechanisms through which palm TRF may alleviate DPN and to identify potential targets for future therapeutic strategies.

## 2. Results

### 2.1. Effect of Palm TRF on Body Weight, Blood Glucose Level, and Relative Organ Weight of Diabetic Rats with DPN

**Figure 1:**
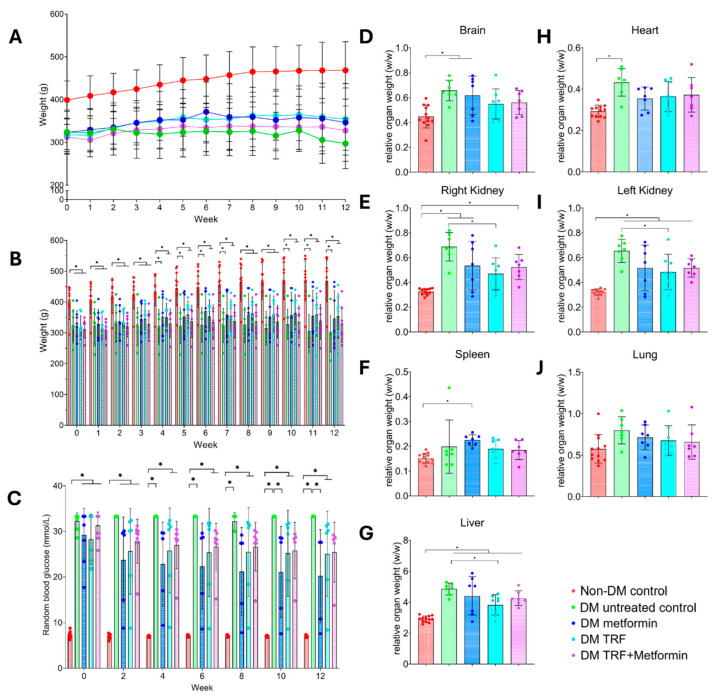
Effects of interventions on body weight, random blood glucose level, and relative organ weight in the non-DM control and DM groups with different treatment interventions from 0- (baseline) to 12-week post-intervention. (**A**) Body weight trends over time during interventions. (**B**) Body weight of each group during the interventions presented with statistical analysis. (**C**) Random blood glucose levels over time during interventions. (**D**–**J**) Relative organ weight of (**D**) brain, (**E**) right kidney, (**F**) spleen, (**G**) liver, (**H**) heart, (**I**) left kidney, and (**J**) lung. Data are presented as mean ± SD. Analysis was performed using one-way ANOVA with Turkey’s post hoc analysis. * *p* < 0.05, where *n* = 8 per group.

A steady increase in body weight was observed in the non-DM control group throughout the 12-week study period, while the DM untreated control group showed a declining trend (Figure 1A and Table S1). At baseline prior to treatment, a significant reduction in body weight was observed in the DM untreated control group (324.0 ± 51.6) compared to the non-DM control group (399.1 ± 44.4), and this significant difference persisted until the end of the treatment period at the 12th week (Figure 1B), consistent with hyperglycaemia-induced catabolism. No significant difference was observed between the DM groups assigned to different treatments at baseline, indicating that the animals were well-randomised before the intervention. After 12 weeks of treatment, the body weight of the DM untreated control group (299.0 ± 69.5) was significantly lower compared to the non-DM control group (472.1 ± 68.7). The DM metformin (332.1 ± 93.4), DM TRF (344.4 ± 76.1), and DM TRF + metformin (331.4 ± 57.1) groups demonstrated a similar trend of body weight increase compared to the DM untreated control group (299.0 ± 69.5) after 12 weeks of intervention.

The random blood glucose levels of the DM untreated control group (32.3 ± 1.8) at the baseline showed a significant increase when compared to the non-DM control group (7.3 ± 0.7) (Figure 1C). Starting from week 10 to week 12 of intervention, the DM metformin group showed a significant reduction in random blood glucose levels compared to the DM untreated control group. A similar trend to the DM metformin group was observed in the DM TRF and DM TRF + metformin groups but no significant difference was observed when compared to the DM untreated control group.

Relative organ weight (*w*/*w*) to body weight are shown in Figure 1D–J. There was a significant increase in relative organ weight in the DM untreated control group compared to the non-DM control group for brain, left and right kidneys, heart, and liver. The DM group treated with TRF showed a significant reduction in the relative weight of both kidneys and liver compared to the DM untreated control group.

### 2.2. Effect of Palm TRF on Mechanical Allodynia and Thermal Hyperalgesia on Diabetic Rats with DPN

#### 2.2.1. Mechanical Allodynia

**Figure 2:**
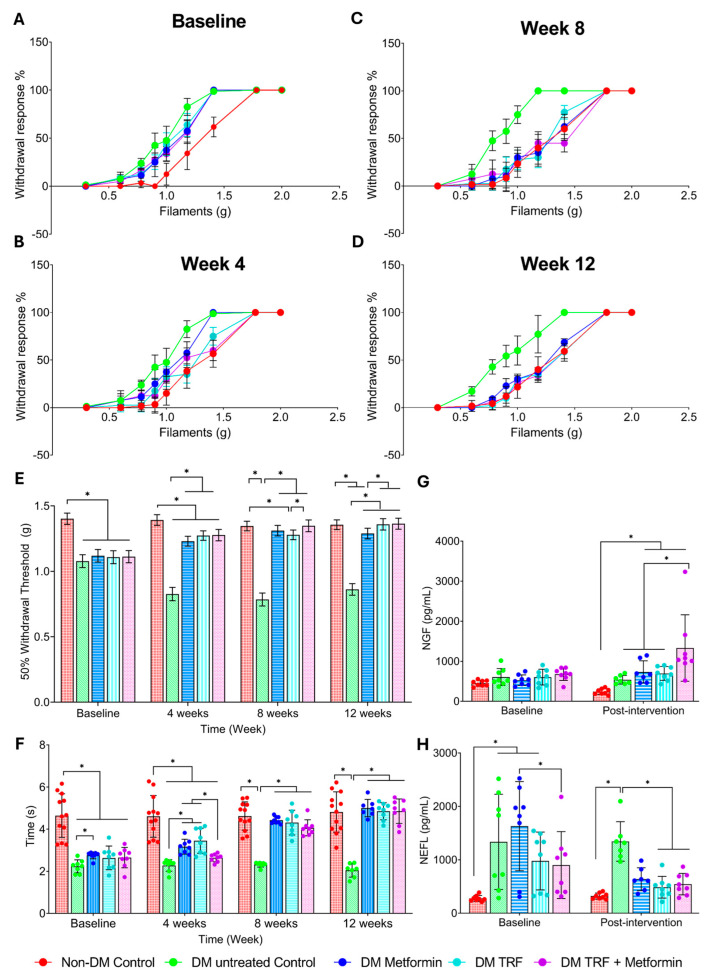
Effect of palm TRF on DPN’s behavioural and blood parameters where (**A**–**D**) percentage withdrawal response in the von Frey filament test at (**A**) baseline, (**B**) week 4, (**C**) week 8, (**D**) week 12, (**E**) 50% withdrawal threshold; (**F**) tail withdrawal time for tail immersion study; (**G**) serum level of nerve growth factor; (**H**) serum level of neurofilament light chain. Data are presented as mean ± SD, and analysis was performed using one-way ANOVA with Turkey’s post hoc analysis. * *p* < 0.05, where *n* = 8 per group.

The percentage of withdrawal response (%) in the von Frey filament test was plotted as a line graph, and trends between different groups were observed at baseline (0), 4, 8, and 12 weeks of treatments (Figure 2A–E). The DM untreated control group’s line remained to the left of the non-DM control group’s line from baseline until the end of the study period (Figure 2A–D). At 4 weeks of treatments, the lines of the DM metformin, DM TRF and DM TRF + metformin groups shifted to the right, toward the non-DM group, positioning between the DM untreated control and non-DM control groups (Figure 2B). At 8 and 12 weeks of treatment, all three treated DM groups shifted further to the right, following a similar trend to the non-DM control group (Figure 2C,D). The 50% withdrawal response values were calculated for statistical analysis (Figure 2E). A significant decrease in the 50% withdrawal response was observed in the DM untreated control group compared to the non-DM control group, from baseline through the 12-week study period, suggesting that combination of high-fat diet and low-dose streptozotocin (STZ) induced persistent pain (mechanical allodynia) in untreated DM group. The 50% withdrawal response increased in the DM groups receiving any of the three treatments (metformin, TRF and TRF + metformin), compared to the DM untreated control group, indicating that all treatments alleviate mechanical allodynia in DM rats (Figure 2E).

#### 2.2.2. Thermal Hyperalgesia

To study the effect of intervention on thermal hyperalgesia in rats with DPN, tail withdrawal latency was recorded at baseline, 4, 8, and 12 weeks (Figure 2F). From baseline until the end of the study period, the DM untreated control group showed a significant decrease in tail withdrawal time compared to the non-DM control group. Tail withdrawal time in both DM metformin and DM TRF groups was significantly increased compared to the DM untreated control group at 4, 8, and 12 weeks of intervention. The DM metformin + TRF group showed a significant increase in tail withdrawal time compared to the DM untreated control only at 8 and 12 weeks of intervention.

### 2.3. Effect of Palm TRF on Serum Nerve Growth Factor (NGF) and Neurofilament Light Chain (NEFL) Levels in Diabetic Rats with DPN

There was no significant difference in NGF levels between the DM untreated control group and the non-DM control group at baseline and after 12 weeks of the study period (Figure 2G). Only the DM TRF + metformin group showed an increase in NGF levels at 12 weeks of intervention compared to the DM untreated group. In contrast, NEFL levels were higher in the DM untreated control group compared to the non-DM control group at baseline and after 12 weeks of the study period (Figure 2H). Interestingly, both DM groups treated with TRF, either alone or in combination with metformin, showed a reduction in NEFL levels compared to the DM untreated group at 12 weeks of intervention. However, the DM groups treated with metformin alone did not show a significant difference compared to the DM untreated group at the end of the study period.

### 2.4. Effect of Palm TRF on the Plasma Metabolomic Profile of Diabetic Rats with DPN

**Figure 3:**
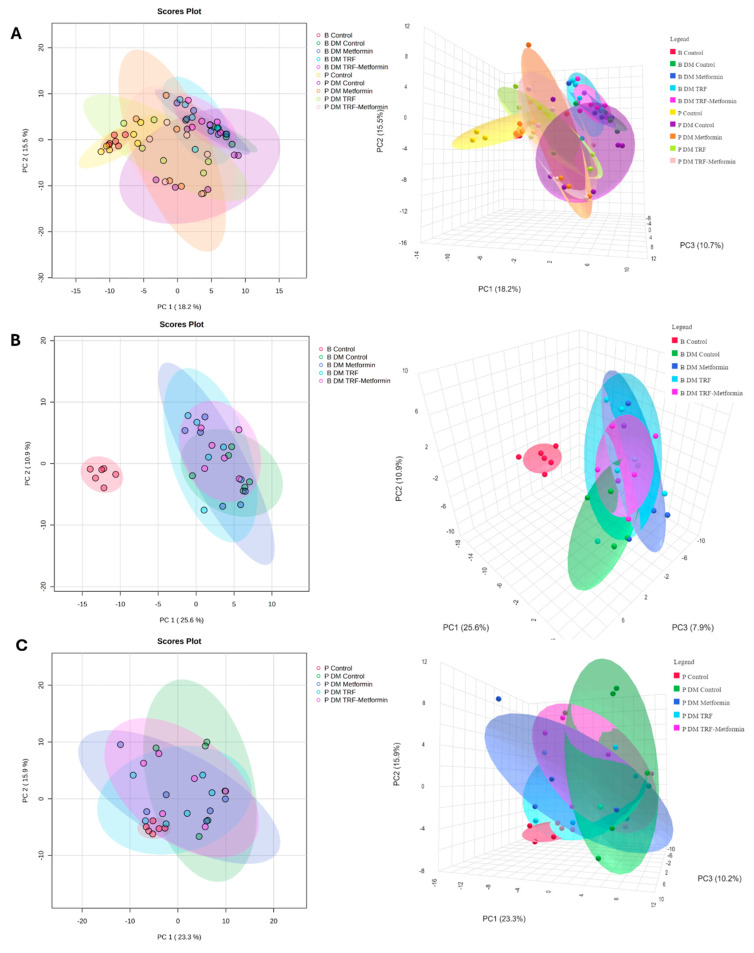
Score plots of PCA for plasma metabolome of non-DM control and DM groups with different interventions at (**A**) baseline and post-intervention, (**B**) all group baseline, and (**C**) all group post-intervention, respectively, *n* = 6 per group. Colours indicate the respective experimental groups, with the left panels representing the 2D PCA views and the right panels showing the corresponding 3D PCA views.

From the plasma samples, a total of 180 metabolites is annotated. Based on the PCA score plots, the plasma metabolomic profiles of all the groups were not well separated, except for the non-DM control group, which showed clear separation, particularly at baseline, from the other groups (Figure 3 and Figures S1–S3). Following 12 weeks of intervention, the treated groups showed a slight tendency to cluster closer to the non-DM group, indicating partial metabolic modulation with TRF and metformin supplementation. A total of 22 differential expressed metabolites (DEMs) were identified among the groups after 12 weeks of intervention (Table 1). These DEMs were identified based on unadjusted *p* < 0.05. After applying False Discovery Rate (FDR) correction using the Benjamin–Hochberg method, four metabolites remained significant (*q* < 0.05), as detailed in Table S3. This finding indicates that several metabolic alterations were robust even after multiple testing correction, supporting their potential biological relevance.

A total of 20 DEMs were observed in the comparison between the non-DM and DM untreated control groups, with half of them upregulated in the DM untreated control group. The top three most upregulated metabolites in the DM untreated group were 4-phenylbutyric acid (73.06-fold), glycocholic acid (42.94-fold), and a bile acid (tauroursodeoxycholic acid, taurochenodeoxycholic acid, or taurodeoxycholic acid) (38.48-fold), while the top three most downregulated metabolites were creatine (3.73-fold), acetyl-beta-methylcholine (2.47-fold), and arginine (2.35-fold). Compared to the DM untreated control group, only two DEMs were identified in the DM metformin group, both of which were upregulated, including metformin itself (6.1-fold) and arginine (2.21-fold). In the DM TRF group, six DEMs were identified relative to the DM untreated control group. These included four upregulated metabolites, namely acetylcholine (2.09-fold), arginine (1.85-fold), 4-oxoproline (1.68-fold), and cytosine (1.6 fold), as well as two downregulated metabolites, i.e., a bile acid (tauroursodeoxycholic acid, taurochenodeoxycholic acid, or taurodeoxycholic acid) (38.83-fold) and a methylguanine (7-methylguanine or 1-methylguanine) (1.4-fold). The DM TRF + metformin group exhibited three upregulated DEMs compared to the DM control group, namely metformin (13.87-fold), cytosine (1.56-fold), and phenylalanine (1.29-fold).

To determine changes in the metabolites between the baseline and post-intervention, a comparison between these two time points in all groups was analysed and the result is shown in Table S2. A total of 28 DEMs out of 180 annotated metabolite features were determined in the non-DM control group when compared between the baseline and post-intervention. In this comparison, 15 metabolites showed an upregulation while 13 metabolites showed a downregulation. Uridine and 3-hydroxybutyric acid were the most upregulated metabolites in this non-DM control group with a 3.08-fold increase and 3.22-fold increase, respectively. The fold change comparison showed that (+/−)9,10-dihydroxy-12Z-octadecenoic acid (2.35-fold decrease) was the most downregulated metabolite in this group when comparing the two time points. For the DM untreated control group, a total of 30 DEMs out of 180 metabolite features were identified when comparing the baseline and post-intervention. A total of 16 metabolites were upregulated, and 14 metabolites were downregulated. Indole was the most prominent metabolite that showed an upregulation with a 7.31-fold increase, while suberic acid showed a downregulation with a 4.26-fold decrease.

For the DM metformin group, 18 metabolites showed an upregulation while 9 metabolites showed a downregulation with a total of 27 DEMs out of 180 metabolic features when comparing the baseline to the post-intervention. From the comparison, catechol and indole were among the most prominent metabolites which showed an upregulation with a 9.69-fold increase and 7.58-fold increase, respectively. On the other hand, N-acetyl-carnosine and suberic acid show a downregulation with a 4.51-fold decrease and a 4.64-fold decrease, respectively. For the TRF intervention, a total of 50 DEMs out of 180 metabolite features were identified when comparing the baseline and post-intervention data. Out of 50 DEMs, 34 metabolites showed an upregulation, and the remaining 16 metabolites showed a downregulation. Among all, indole as the most prominent upregulated metabolite showed a 5.08-fold increase, while the most prominent downregulated metabolite is taurodeoxycholic acid with a 4.33-fold decrease. For a combination of TRF and metformin (DM TRF + metformin), a total of 39 DEMs were detected where 29 metabolites showed an upregulation, while 10 metabolites showed a downregulation. Catechol showed the most upregulation with a 10.11-fold increase, while 4-phenylbutyric acid showed a 3.60-fold decrease when comparing the two time points.

### 2.5. Pathway Analysis of the DEMs Following Palm TRF Intervention in Diabetic Rats with DPN

**Figure 4:**
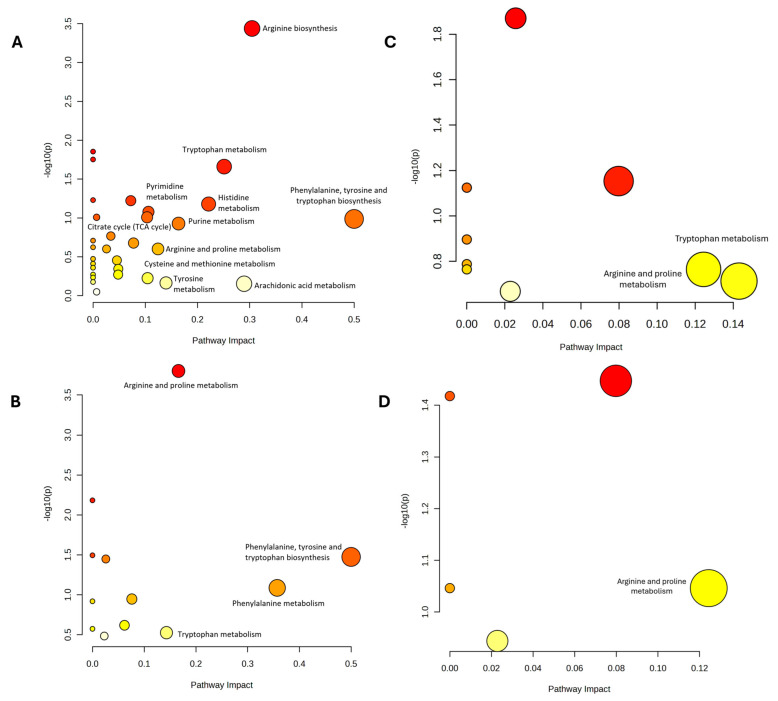
The pathway analysis (**A**–**D**) on the DEMs of plasma sample between (**A**) baseline and post-intervention for each group (**B**) All group at 12 week, (**C**) DMC vs. control, (**D**) DMT vs. DMC, *n* = 6 per group. Bubble size indicates pathway impact, while bubble color reflects the level of statistical significance.

The DEMs identified from both time-point comparisons within each group as well as between-group comparisons at post-intervention were subjected to pathway analysis. In the within-group time-point comparisons across all groups, two impactful and significant pathways were identified, phenylalanine, tyrosine, and tryptophan biosynthesis; and arginine biosynthesis (Figure 4A, Table 2). For the overall between-group comparison at the post-intervention time point, two impactful and significant pathways were identified, including phenylalanine, tyrosine, and tryptophan biosynthesis; and arginine and proline metabolism (Figure 4B, Table 2).

Specifically, the two-group comparison between the non-DM and DM untreated control groups revealed two impactful pathways which include tryptophan metabolism; and arginine and proline metabolism (Figure 4C, Table 2). In the comparison between the DM TRF and DM untreated control groups, only one impactful and significant pathway was identified which is arginine and proline metabolism as shown in Figure 4D and Table 2.

### 2.6. Correlation Analysis Between the Plasma Metabolites and the DPN’s Behavioural Parameters and Serum Neuronal Biomarkers

**Figure 5:**
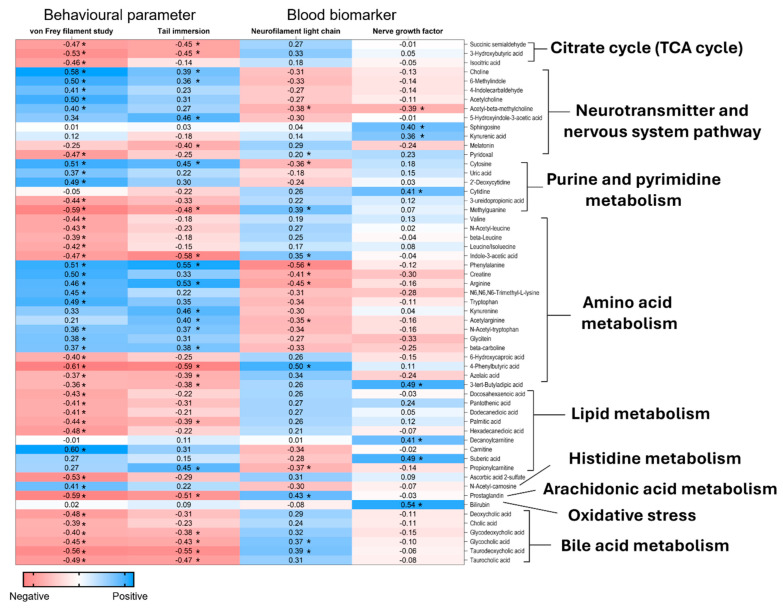
Heatmap of metabolites with significant correlation coefficient (*r*) with behavioural and blood parameters in DPN rats. * *p* < 0.05.

Correlation analysis between all annotated metabolites and the DPN-related behavioural parameters and serum neuronal biomarkers at post-intervention revealed mixed trends (Figure S4). Metabolites with significant correlation coefficient (*r*) in relation to DPN findings are presented in Figure 5. A positive correlation to the DPN behavioural parameters indicated that with increasing concentration of the metabolites, the time taken for the pain sensation is also increasing, thus reducing the pain sensitivity and the DPN symptoms. For instance, metabolites associated with neurotransmitter and nervous system pathways, such as choline, acetylcholine, and acetyl-beta-methylcholine, showed positive correlations with von Frey filament study but negative correlations with serum NEFL levels. Choline also showed a significant positive correlation in the tail immersion study while acetyl-beta-methylcholine also showed a negative significant correlation to the NGF.

In contrast, metabolites related to the TCA cycle, including succinic semialdehyde and 3-hydroxybutyric acid, were negatively correlated with both behavioural parameters, while isocitric acid only showed a significant negative correlation to von Frey filament study. Metabolites involved in the amino acid metabolism, including phenylalanine and arginine, showed significant positive correlation to both behavioural parameters with significant negative correlation to NEFL. Additionally, metabolites involved in lipid and bile acid metabolisms, including docosahexaenoic acid, dodecanedioic acid, palmitic acid, cholic acid, glycodeoxycholic acid, glycocholic acid, taurocholic acid, and taurodeoxycholic acid-related metabolite, exhibited negative correlations with von Frey filament study but positive correlation with NEFL levels. Notably, prostaglandin, a metabolite from the arachidonic acid pathway, also demonstrated a negative correlation with behavioural parameters and a positive correlation with NEFL levels.

## 3. Discussion

In this study, we investigated the effects of palm TRF on the DPN-related behavioural parameters and the two related blood biomarkers before an untargeted metabolomic analysis was performed on the plasma samples. The observed weight loss in type II DM rats reflects the metabolic consequences of chronic hyperglycaemia, including increased protein and fat catabolism, osmotic diuresis, and reduced nutrient utilisation [31,32]. Importantly, the effects of TRF and metformin on neuropathic parameters were assessed relative to the diabetic control group, indicating that treatment effects are independent of body weight differences. In addition, the stabilisation of body weight in treated diabetic rats suggests that TRF and metformin may mitigate some of the catabolic effects associated with chronic hyperglycaemia, whereas untreated diabetic rats continue to lose weight due to persistent metabolic dysfunction, including protein and fat catabolism and osmotic diuresis.

### 3.1. Behavioural Assessment

For the behavioural parameter, palm TRF treatment improved neuropathic symptoms by increasing the 50% withdrawal threshold and tail withdrawal latency, indicating reduced mechanical allodynia and thermal hyperalgesia in diabetic rats. Antioxidants such as ferulic acid, alpha-lipoic acid, syringic acid, and vitamin E have been proven to improve hyperalgesia and allodynia in DPN animal models [33,34,35]. Palm TRF, rich in tocotrienols and containing a smaller proportion of tocopherols, represents a potent component of vitamin E with more antioxidant properties compared to α-tocopherol alone [36]. Palm TRF has various medicinal properties, including enhancing cognitive functions, reducing oxidative stress, and neuroinflammation, and it has recently shown potential benefits against neurodegenerative disorders such as Alzheimer’s disease [16,37]. Antioxidant and neuroprotective properties of this palm TRF might explain the improvement in the hyperalgesia and allodynia found in the DM group treated with TRF. Metformin has also been shown to reduce TNF-α levels and improve hyperalgesia and allodynia in DPN animals, supporting its neuroprotective role. Clinical studies further showed that metformin users had better neuropathy scores, nerve function, and axonal excitability, likely due to improved Na^+^ and K^+^ conductance [27,38]. This improvement in the DPN symptoms was also noted when the metformin was co-administered with palm TRF and might serve as a safe supplementation to be given to DM patients with concurrent metformin treatment.

### 3.2. Serum Neuropathy Biomarkers

To further elucidate the effects of metformin and palm TRF on DPN, serum biomarkers were evaluated. NEFL and NGF were selected as they serve as important indicators of neuronal damage, axonal integrity, and neurodegeneration in DPN [39,40]. In our study, rats with DPN showed an increase in the level of NEFL seen at the baseline and the level was reduced after 12 weeks of treatment with TRF, metformin, or a combination of both. This NEFL is one of the three major neurofilament subunits, besides neurofilament medium and neurofilament heavy chain [41]. They are essential structural components of axons, responsible for maintaining axonal integrity and stability, which are crucial for normal neuronal function and signal transmission [42]. The NEFL is released into the cerebrospinal fluid and blood following axonal injury, and elevated levels of NEFL have been reported as a potential biomarker in DPN, indicating axonal damage and impaired nerve conduction. In contrast, nerve growth factor (NGF) plays an important role in promoting neural regeneration and survival in both the central and peripheral nervous systems [41]. This NGF can promote regeneration of the neuron, tissue remodelling and functional recovery in spinal cord injury [43]. In this study, treatment with TRF and metformin did increase the level of NGF in the rats with DPN and a combination of TRF and metformin showed a higher level of NGF. Our study showed that intervention with palm TRF might improve the symptoms of DPN as seen in behavioural parameters due to the reduction in neuronal damage or increase in nerve regeneration.

### 3.3. Untargeted Metabolomic Profiling

Network analysis was performed in MetaboAnalyst 6.0 to identify the biochemical relationships and potential functional linkages among the differential metabolites as shown in Figure S6. The generated metabolite–disease interaction network demonstrated several highly connected nodes, revealing metabolites that may serve as key metabolic hubs in DPN. Untargeted metabolomic analysis of plasma identified DEMs associated with DPN, showing features of type II DM, obesity, mitochondrial dysfunction, and metabolic syndrome with neurological complications. These results indicate that DPN rats exhibit metabolic alterations, consistent with type II DM and metabolic syndrome, supporting the role of metabolic dysregulation beyond hyperglycaemia in DPN development [44,45,46].

**Figure 6:**
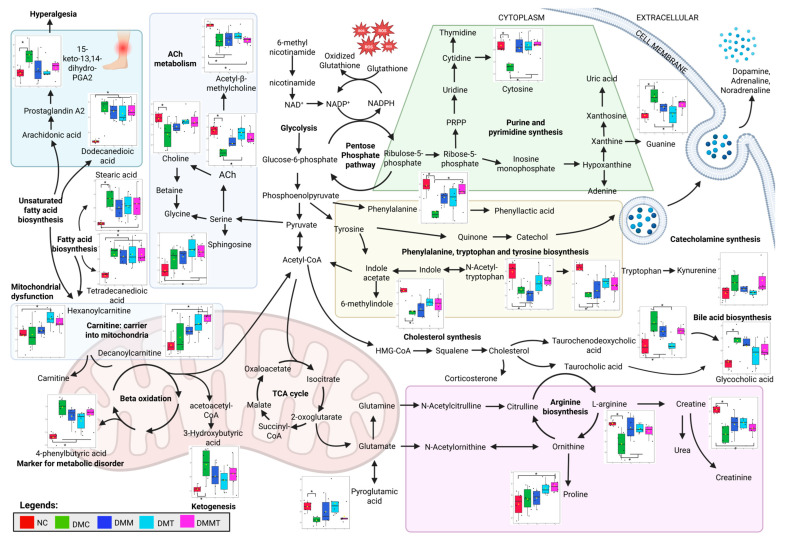
Integrated biochemical pathways altered in the non-DM control (NC) and DM groups with different treatments after 12 weeks of intervention. Differential expressed metabolites (DEMs) are presented in boxplots. Analysis was performed using one-way ANOVA with Turkey’s post hoc analysis. * *p* < 0.05, where *n* = 6 per group. Created in Biorender, Rusli, N. (2025) https://app.biorender.com/illustrations/663e9f24013d4f49abe2c210?slideId=6ddf030d-aa30-4ee7-a5d2-774a32c4190a, accessed on 12 September 2025.

In this study, both unadjusted and FDR-adjusted *p* values were considered to balance statistical stringency with biological relevance. While only a subset of metabolites remained significant following FDR correction, the consistent patterns observed across both analyses suggest that these metabolic changes are biologically meaningful. Given the exploratory nature of this untargeted metabolomic approach, presenting both adjusted and unadjusted results provides a comprehensive overview of metabolic alterations associated with DPN and treatment responses. Thus, to further understand the complex relationship between this metabolic syndrome, type II DM, and DPN, an integrated pathway with a summary of significantly differentially expressed metabolites (DEMs) is illustrated in Figure 6 and Figure S5, where each of the DEMs is presented in boxplots. In this study, the metabolite profiles between each group were compared to provide insight into the metabolites affected by DPN, which are regulated back by palm TRF and metformin. DPN rats showed elevated fatty acids and their related metabolites, reflecting mitochondrial dysfunction and incomplete β-oxidation, which may contribute to DPN progression [47,48,49,50]. Fatty acids and lipid-related metabolites were elevated in DPN rats and showed strong negative correlations with the behavioural parameters. This indicates that higher levels of fatty acids were associated with shorter withdrawal latencies, reflecting increased pain sensitivity manifested as enhanced hyperalgesia and allodynia.

Interestingly, in this study, the level of 13,14-dihydro-15-keto prostaglandin A_2_, a degradation product of prostaglandin, was elevated in the DPN rats as compared to the non-DM control rats, likely reflecting dysregulation of fatty acid biosynthesis. While parent prostaglandins like prostaglandin E_2_ (PGE_2_) and prostaglandin I_2_ (PGI_2_) are the key mediators of hyperalgesia via the sensitization of nociceptors, 13,14-dihydro-15-keto prostaglandin A_2_ is formed during the degradation and inactivation of active prostaglandins [51]. PGI_2_ is responsible for vasodilation while PGE_2_ is responsible for both vasodilation and pain perception [52]. Vasodilation enhances blood flow to the skin, facilitating the delivery of pro-inflammatory cytokines, which subsequently trigger and amplify the inflammatory response. These conditions might explain the symptoms of hyperalgesia seen in the DPN rats through the tail immersion study. In this study, prostaglandin metabolite, 15-keto-13,14-dihydro-prostaglandin A2, was elevated in DPN rats when compared to the other groups, which may contribute to the development of hyperalgesia and allodynia. In contrast, palm TRF and metformin levels reduced the level of this metabolite, correlating with improved behavioural outcomes (prolonged withdrawal latency) and lower NEFL.

Carnitine plays a crucial role in mitochondrial function, fatty acid oxidation, and metabolic flexibility, while also protecting against oxidative stress and supporting both ketogenesis and glucogenesis. Deficiency in carnitine has been linked to a range of metabolic and non-metabolic disorders, including diabetes, liver, kidney, endocrine, and organic acidurias conditions [53,54,55]. In addition, L-carnitine supplementation has been shown to improve insulin resistance and restore mitochondrial function in high-fat diet-induced obesity models by inducing autophagy through peroxisome proliferator-activated receptor gamma (PPARγ) activation [56]. In this study, carnitine levels positively correlated with the von Frey withdrawal response, indicating that higher carnitine reduces allodynia symptoms in DPN rats. This finding is supported by another study which found that carnitine has neuroprotective, neurotrophic, and antioxidant effects that show promise in treating neurodegenerative diseases [57].

Treatment with palm TRF, either alone or in combination with metformin, increased the levels of hexanoylcarnitine and decanoylcarnitine after 12 weeks compared to baseline (Figure S5). These changes indicate enhanced mitochondrial fatty acid transport and β-oxidation, which may contribute to the restoration of energy metabolism through the TCA cycle. A study conducted on TCM JinMaiTong showed improved glycolysis and TCA cycle function and alleviated DPN symptoms [29]. In our study, within-group comparison showed that DPN rats had a reduced level of 2-oxoglutarate or α-ketoglutarate, as shown in Figure S5, indicating progressive disruption of the TCA cycle in the absence of intervention. In addition, we also find that there is an elevated level of 4-phenylbutyric acid in the DPN rats, which is naturally upregulated as part of an adaptive response to ER stress and may help mitigate inflammation and oxidative stress [58]. Intervention with palm TRF surprisingly reduced the level of 4-phenylbutyric acid, which indicates that there is a reduction in inflammation and oxidative stress in the DPN rats.

The ketogenesis marker, 3-hydroxybutyric acid, along with TCA cycle metabolites (succinic semialdehyde and isocitric acid), showed negative correlations with behavioural parameters, highlighting their potential roles in metabolic dysfunction in DPN [59]. In mitochondrial dysfunction, accumulation of isocitrate, and citrate with a reduction in the level of α-ketoglutarate and succinate are normally seen, and this is also seen in the DPN rats in our study [60,61]. In our study, we find that supplementation with palm TRF also increased the level of 13-hydroxyoctadecadienoic acid (13-HODE) and 9,11-dihydroxy-12-octadecenoic acid (9,11-DiHOME), as shown in Figure S5. 13-HODE and 9,11-DiHOME are metabolites of linoleic acid and play a role in activating the PPARγ pathways, which are important in regulating genes that are involved in lipid metabolism, glucose homeostasis, inflammation, and cellular differentiation and survival [62]. PPARγ is predominantly expressed in adipose tissue, macrophages, and neuronal tissues, where its activation regulates neuronal differentiation, axon polarity, and neuroprotection through the reduction in neuronal death and inflammation, pathways that are relevant to the pathophysiology of DPN [63,64,65].

From our study, the level of choline and acetylcholine (Ach) were reduced in the DPN rats, and intervention with palm TRF increased the level of both metabolites either alone or in combination with metformin. Choline showed a positive correlation with both behavioural parameters, while ACh showed a positive correlation only to von Frey filament study. ACh is synthesised in the presynaptic neuron from choline and acetyl-CoA which is derived from the energy metabolism [66]. In the peripheral nervous system, Ach plays a crucial role by activating muscarinic receptor. Experimental studies also suggest that modulation of muscarinic receptor activity may exert neuroprotective effects in DPN models [67]. In the present study, supplementation with palm TRF restored the levels of choline and acetylcholine, which were associated with improvements in thermal hyperalgesia and mechanical allodynia, as reflected by higher 50% withdrawal response and longer tail withdrawal latency in DPN rats. These observations imply a relationship between cholinergic metabolism and neuronal integrity in DPN rats treated with TRF. From our study, the level of catechol is increased in the DPN rats treated with metformin, palm TRF, and a combination of both. This catechol is important for catecholamine synthesis such as dopamine, adrenaline, and noradrenaline [68]. This catechol is synthesised from tyrosine and is part of phenylalanine, tryptophan, and tyrosine metabolism. All the metabolites in this pathway, including 6-methylindole, phenylalanine, N-acetyl-tryptophan, and tryptophan, are downregulated in the DPN rats, and this was reversed with supplementation of palm TRF, as seen from Figure 6. N-acetyl-tryptophan, phenylalanine, and 6-methylindole showed a positive correlation with both behavioural parameters, while tryptophan only showed a positive correlation with von Frey filament study. However, only phenylalanine showed a negative correlation with the NEFL.

In summary, the overall pathways affected in DPN and following treatments are summarised in Figure 6. Integration of plasma untargeted metabolomics with behavioural and blood biomarker correlations identified key metabolites and pathways that were altered in DPN and were responsive to palm TRF supplementation. Overall, the improvements in behavioural responses and normalisation of biochemical markers observed in the TRF-treated rats were consistent with the metabolomic alterations identified, collectively supporting the protective role of TRF in modulating oxidative and inflammatory pathways underlying DPN. Future studies should explore nerve-ending metabolomics alongside IENFD staining to better elucidate the early pathogenesis of DPN and identify potential therapeutic targets. Investigating the molecular mechanisms of specific pathways may further enhance our understanding of DPN development, ultimately guiding the identification of effective strategies for its prevention and treatment.

## 4. Materials and Methods

### 4.1. Animal Model and Treatment with Palm Tocotrienol-Rich Fraction

Healthy male Sprague Dawley (SD) rats (*n* = 46) aged 12 weeks (weight 200–250 g) were purchased from the Laboratory Animal Resource Unit (LARU), Universiti Kebangsaan Malaysia. The sample size was determined based on previous studies on diabetic peripheral neuropathy (DPN) models, which demonstrated that this number was sufficient to detect significant differences in behavioural, biochemical, and histological outcomes [29]. Throughout the study, the rats were randomly maintained in Sealsafe^®^ Plus Rat IVC Green Line (Techniplast, Buguggiate, Italy) at a temperature of 24 ± 2 °C, with a 12 h light–dark cycle, and were provided with normal rat pellets and water ad libitum. All animal-handling procedures were approved by the Universiti Kebangsaan Malaysia Animal Ethics Committee (UKMAEC) on 18 February 2022, under approval code FP/2022/RABANI/26-JAN./1229-JAN.-2022–SEPT.-2024, with study duration between 10 March 2022 and 9 March 2025. The rats were randomly divided into a non-DM control group (*n* = 8) and a DM group (*n* = 38) and were acclimated for a week. The DM group was then given a high-fat diet (HFD), while the non-DM control group was given normal rat pellets during the induction phase of DPN. In week 2 after the introduction of HFD, a low dose of streptozotocin (STZ) (30 mg/kg) was administered via intraperitoneal (IP) injection [69,70]. Random blood glucose was measured from the tail vein using a glucometer 72 h post-STZ injection to confirm the development of Type II DM in the rats [71]. Rats with a blood glucose level of more than 13.8 mmol/L were considered diabetic and were included in the study (*n* = 33). Rats that were not diabetic (*n* = 5) were excluded from the study. Even though diabetes was already established in the animal model, the animals were continuously supplemented with a high-fat diet throughout the experiment to maintain Type II DM conditions in the model. Random blood glucose was monitored in both the non-DM control and DM groups every two weeks, while body weight was monitored every week.

**Figure 7:**
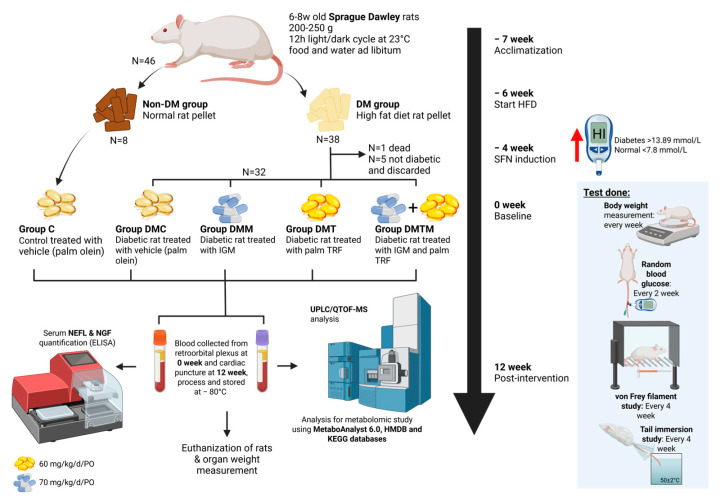
Schematic presentation of experimental design and procedures starting with the acclimatization until animal euthanization and organ collection. Created in BioRender. Rusli, N. (2025) https://app.biorender.com/illustrations/67d26dea3ba502dc0e54452a?slideId=58228c2a-7229-4bc8-ac21-86f6291faa19, accessed on 12 September 2025. Abbreviations: C: non-DM control; d: day; DM: diabetes mellitus; DMC: diabetes mellitus untreated control; DMM: diabetes mellitus metformin; DMT: diabetes mellitus TRF; DMTM: diabetes mellitus TRF metformin; HFD: high-fat diet; HMDB: Human Metabolite database; ELISA: enzyme-linked immunosorbent assay; IGM: intragastric metformin; KEGG: Kyoto Encyclopaedia of Genes and Genomes; kg: kilogram; mg: milligram; N: biological replicate; NEFL: neurofilament light chain; NGF: nerve growth factor; PO: per oral; TRF: tocotrienol-rich fraction; UPLC/QTOF-MS: Ultra-Performance Liquid Chromatography–Quadrupole Time-of-Flight Mass Spectrometry.

After DM induction, rats in the DM group (*n* = 32) were further divided into 4 groups randomly, consisting of DM untreated control, DM metformin, DM TRF, and DM TRF + metformin (*n* = 8 per group), as shown in Figure 7 and are considered as the baseline. The TRF used in this study contained 24% α-tocopherol, 27% α-tocotrienol, 4% β-tocotrienol, 32% γ-tocotrienol, and 14% δ-tocotrienol, while the vehicle used in this study was refined (RBD) palm olein. Both TRF (Golden Tri™ E 70) and RBD palm olein were generously provided by Sime Darby Plantation Berhad. The TRF treatment (60 mg/kg of body weight) was prepared weekly by dissolving TRF in palm olein under dark conditions and was kept at 4 °C, wrapped in aluminium foil to protect it from sunlight. For metformin preparations, a dose of 70 mg/kg of body weight was dissolved in sterile distilled water. Both TRF and metformin were administered via oral gavage daily for 12 weeks.

### 4.2. Neuropathic Behaviour Study

#### 4.2.1. Von Frey Filament Study

Mechanical allodynia was assessed using the von Frey filament study at baseline, 4 weeks, 8 weeks, and 12 weeks of intervention, with slight modification from the previous study [72]. The rats were randomly placed individually into a ten-compartment rat enclosure with wire mesh floors and lids. To minimise exploratory activity, the rats were allowed a 20 min habituation period. A 2 g filament (North Coast Medical Inc., Morgan Hill, CA, USA) was first applied to the bilateral hind paws with sufficient force to buckle the filament slightly for a maximum of 6 s. Any response, either immediately or within 6 s, such as licking, flinching or withdrawing from the filaments, was considered a positive response. If no such response occurred during the application, a negative response was recorded. This procedure was repeated 5 times for each filament, and the test was continued with a bigger filament number (4 g, 6 g, 8 g, 10 g, 15 g, 26 g, 60 g, 100 g and more) until five positive responses were obtained for two consecutive filaments. The response for each attempt with each filament was then calculated as the percentage (%) withdrawal response using the formula as follows:

$$Percentage\;\left(\%\right)\;withdrawal\;response\;=\;\frac{Number\;of\;positive\;responses}{Number\;of\;applications}\;\times \;100$$

By using GraphPad Prism 9.0, a nonlinear regression curve was plotted using the percentage withdrawal response against the filaments, and the log EC_50_ value was obtained. The average measurement for both legs was then calculated and tabulated. A lower withdrawal response indicates symptoms of mechanical allodynia which are normally seen in patients with SFN.

#### 4.2.2. Tail Immersion Study

The tail immersion study was performed to observe thermal hyperalgesia at baseline, 4 weeks, 8 weeks, and 12 weeks of intervention. The temperature of the water bath was set to 50 °C, and a thermometer was used to validate the temperature. The rats were given a 20 min habituation period and were randomly restrained in a plastic restrainer. After acclimating to the surroundings, the tail of the rat was immersed in hot water, and the tail withdrawal latency was recorded. The procedure was repeated to obtain three readings, and for each reading, a cutoff time of 30 s was set to prevent tissue damage and injury [73]. A shorter latency indicates symptoms of thermal hyperalgesia, which are normally shown as increased sensitivity towards pain sensation [74].

### 4.3. Blood Collection

Blood was collected from the retroorbital plexus using a capillary tube at baseline and post-treatment. The rats were anaesthetized before blood collection, and a total of 3 mL of blood was collected into EDTA and plain tubes before gently removing the capillary tube. The bleeding was stopped by applying gentle pressure with a finger and sterile cotton. The blood was kept on ice and separated by centrifugation at 3000 rpm for 15 min. Both the plasma and serum were aliquoted into several tubes for respective experiments and stored at −80 °C for further analysis.

### 4.4. Euthanization of Animal and Organ Collection

For this study, KTX agents, which are a combination of ketamine, xylazine, and zoletil-50 (tiletamine and zolazepam), were used as anaesthetic agents. The rats were fasted overnight with water available ad libitum. The KTX agents were administered at a dose of 0.1 mL/100 g of rat body weight to each rat by intravenous injection into the tail vein. The rats were left for approximately 30 min for KTX agents to exert their sedative and anaesthetic effects. The animal was considered sedated once it showed clinical signs such as disorientation, loss of consciousness, depression of respiration or rapid irregular breathing, progressively declining heart rate and blood pressure, urination, and defecation. Later, the rats were euthanised via the decapitation method using a decapitator (Modiezham Sdn. Bhd., Kuala Lumpur, Malaysia). Once euthanised, various organs of the SD rats, such as the heart, liver, kidneys, brain, and skin in the thigh area, were dissected for further analysis. All organs were washed with 0.9% normal saline to remove any adherent tissue and weighed. The organ weights were measured as quickly as possible to avoid drying, and they were analysed relative to the body weight of the animals.

### 4.5. Serum Biomarkers Assessment

The levels of neurofilament light chain (NEFL) as a potential neuronal damage marker and nerve growth factor (NGF) as a neuronal growth marker in the serum of the rats were determined using a sandwich enzyme-linked immunosorbent assay (ELISA) kit (ELK Biotechnology CO., Ltd., Wuhan, China) [75,76]. Using the optimised sample dilution factor, 100 μL of the serum sample was added to the wells, and the ELISA was performed following the manufacturer’s protocols. A standard curve ranging from 15.63 to 1000 pg/mL was plotted, and the concentration of NEFL and NGF in the samples was calculated based on the optical density and the standard curve. The concentration of the biomarkers was then plotted on the graph.

### 4.6. Plasma Sample Preparation for Metabolite Extraction

Reagents used for metabolomic analysis were MS-graded from Fisher Scientific (Hampton, NH, USA). The metabolite extraction was performed according to a previous study [77]. Briefly, prior to the analysis of the metabolites, the plasma was thawed on ice and 200 μL was aliquoted into a 2 mL tube. Cold 100% methanol (600 μL) was then added to the tube at a ratio of 1:3, and the mixture was vortexed for 30 s. The mixture was then centrifuged at 16,000× *g* for 15 min at 4 °C. The supernatant was transferred into a new 2 mL tube for drying using a speed vacuum concentrator (Eppendorf, Hamburg, Germany). The dried extracted metabolites were then stored in a −80 °C freezer.

### 4.7. Metabolomic Data Acquisition by UHPLC-MS/MS

Data acquisition using LC-MS/MS was performed according to a previous study [77]. The samples were assigned coded numbers prior to analysis to conceal group identity and ensure objectivity in the analytical process. To run the metabolomic analysis, the dried extracted sample was reconstituted with 200 μL of 5% methanol. The reconstituted sample was then filtered through a 0.2 μm regenerated cellulose membrane syringe filter (Phenomenex, Torrance, CA, USA) into a vial with an MS glass insert (Phenomenex). The reconstitution buffer was used as a blank. A quality control (QC) sample was prepared by pooling an aliquot of 10 μL from each sample into a tube and transferring it into an MS vial. The QC sample was injected 5 times at the beginning, once every 5 plasma samples, and once at the end of the run. Plasma samples within QC intervals consisted of a representative from each experimental group randomised using the Excel function.

The data were acquired using a UHPLC system (Dionex Ultimate 3000; Thermo Scientific, Waltham, MA, USA) coupled to an Orbitrap-MS (Q Exactive HF, Thermo Scientific) through a heated electrospray ionisation probe. Prior to the run, the instrument was calibrated with Pierce LTQ ESI Positive and Negative Ion Calibration Solutions (Thermo Scientific). A C18 column (100 mm length × 2.1 mm diameter with a 1.7 μm particle size; Synchronis, Thermo Scientific) was used as the stationary phase. The mobile phase A consisted of water with 0.1% formic acid, while the mobile phase B comprised acetonitrile (ACN) with 0.1% formic acid. The column temperature was maintained at 55 °C, while the autosampler was set at 10 °C. All samples were injected at 2 μL per ionisation mode with a flow rate of 0.45 mL/min. A 22 min elution gradient was set as follows: 0.5% B for 1 min; 0.5 to 99.5% B over 15 min; 99.5% B for 4 min; 99.5 to 0.5% B in 0.1 min; and 0.5% B for 1.9 min. Negative ionisation mode was initiated after data acquisition of all the samples in positive mode was completed. The MS settings were tuned as follows: sweep gas flow rate at 50 arbitrary unit (AU), auxiliary gas flow rate at 18 AU, sweep gas flow rate at 0 AU, capillary temperature at 320 °C, S-lens level at 55 AU, auxiliary gas heater temperature at 300 °C, and spray voltage at 3.5 kV (positive mode) or 3.0 kV (negative mode). MS spectra were acquired using a full MS1 scan followed by a data-dependent tandem mass spectrometry (ddMS2) scanning mode as implemented in Xcalibur 4.0 software (Thermo Scientific). Full MS1 scans were collected at a resolution of 60,000 with a scan range of 100–1000 *m*/*z*, while ddMS2 scans were collected at a resolution of 15,000 with stepped normalised collision energies of 20, 40, and 60 AU.

### 4.8. Metabolomic Data Processing and Statistical Analysis

The raw data for the UHPLC-MS analysis was processed for peak detection, alignment, blank subtraction, and annotation using Compound Discoverer 2.0 software (Thermo Scientific). Briefly, the minimum intensity threshold for reporting a peak was set at 2,000,000 as recommended by the software. Peak alignment was performed within a mass tolerance of 5 parts per million (ppm), grouping peaks by molecular weight and retention time. Missing peaks within a compound group were imputed using the “Fill Gap” node in Compound Discoverer, which estimates the missing area based on “expected peak width and maximum spectrum noise in the expected retention time range multiplied by the sample/blank (S/N) threshold” as described in the software user guide. Blank peaks were excluded if the S/N ratio was below 5. Metabolite features (MFs), including molecular weight, retention time, and peak intensity, were exported as a comma-separated values (CSV) file for further analysis. A coefficient of variation (CV) cutoff of <30% for QC samples was considered acceptable.

Metabolite annotation was performed automatically by Compound Discoverer using the mzCloud database (HighChem LLC, Bratislava, Slovakia), which was integrated into the software. Only annotated metabolites with level 2 confidence [78] were subjected to statistical analysis. In this study, level 2 confidence annotation was defined as a putative compound matching two orthogonal parameters: accurate mass (<5 ppm) and MS/MS spectral similarity (≥70%) compared to the database. Annotated or duplicated spectra were further manually inspected by the author (TJK) by examining MS1 and MS2 spectra, isotopic patterns, and peak intensities.

The processed data were then further analysed for statistical analysis using MetaboAnalyst 6.0 (https://www.metaboanalyst.ca/, accessed on 15 July 2025). Data were filtered using the interquartile range, while normalisation was performed using log transformation and autoscaling. Principal component analysis (PCA) was performed as part of multivariate data analysis to assess overall differences between the groups. To identify differentially expressed metabolites (DEMs), a univariate analysis using ANOVA was performed, with Tukey’s HSD test as a post hoc analysis. The fold change between each comparison was also calculated. A statistical significance threshold of *p* < 0.05 was applied in this analysis. Although false discovery rate (FDR) correction was considered, it was found to be overly conservative for this dataset, which comprised 180 annotated metabolites (Table S3). For pathway analysis, pathways were identified as significant and impactful when a *p* value of <0.05 and an impact score of >0.1 were reached.

To further confirm the significant correlation between the metabolites and the DPN parameters at *p* < 0.05, a Pearson correlation analysis was performed for normally distributed data, while a Spearman correlation analysis was used for non-normally distributed data. Statistical analysis for other parameters was performed using GraphPad Prism software version 9 (GraphPad Software, San Diego, CA, USA). The distribution of the data was assessed using the Shapiro–Wilk normality test.

## 5. Conclusions

**Figure 8:**
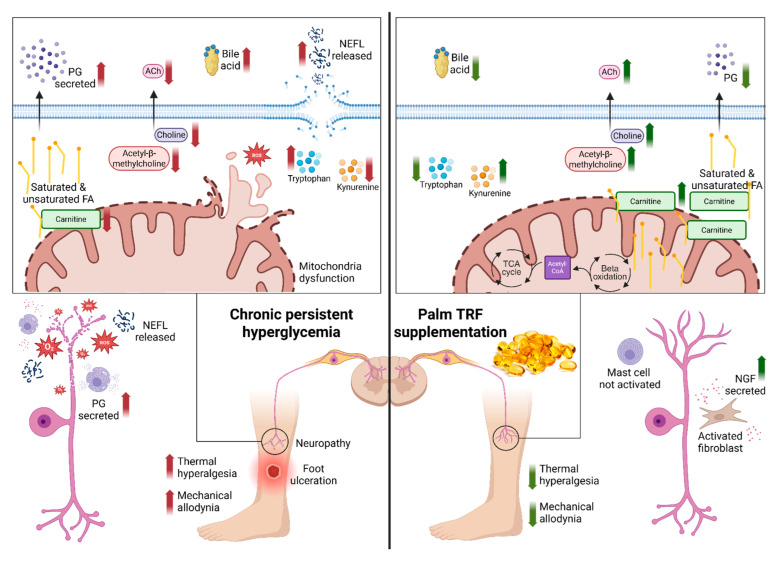
Proposed mechanism of action of tocotrienol-rich fraction (TRF) in mitigating diabetic peripheral neuropathy (DPN) via modulation of mitochondrial function, lipid metabolism, and oxidative stress. The **left** panel illustrates the pathological state in DPN, characterised by increased reactive oxygen species (ROS), reduced carnitine transport into mitochondria, impaired β-oxidation, reduced acetylcholine (ACh) synthesis, and mitochondrial dysfunction. These alterations contribute to nerve degeneration and heightened pain sensitivity. The **right** panel demonstrates the restorative effects of TRF supplementation, including enhanced carnitine uptake, restored β-oxidation and TCA cycle activity, improved ACh synthesis, reduced oxidative stress, and improved mitochondrial function. These changes culminate in nerve regeneration and symptom improvement. Created in Biorender, Rusli, N. (2025) https://app.biorender.com/illustrations/68411e3b50e391347a302613?slideId=42abd94b-db82-4ae2-a669-354c750d10b2, accessed on 12 September 2025. Abbreviation: ACh: acetylcholine; FA: fatty acids; NEFL: neurofilament light chain; NGF: nerve growth factor; PG: prostaglandin; ROS: reactive oxygen species; TCA: tricarboxylic acid; TRF: tocotrienol-rich fraction.

Our study showed that pathways related to neurotransmitters, including acetylcholine metabolism and phenylalanine, tryptophan, and tyrosine biosynthesis as well as pathways associated with energy metabolism such as fatty acid synthesis, arginine biosynthesis, and the TCA cycle, were altered in DPN rats. Intervention with palm TRF, either alone or in combination with metformin, restored the normal regulation of these pathways, as summarised in Figure 8. These metabolic improvements were accompanied by enhanced behavioural outcomes, demonstrated by prolonged withdrawal latency and increased nociceptive thresholds and by reduced serum NEFL levels, indicating attenuation of nerve injury. However, nerve growth factor (NGF) did not show strong correlations with the metabolites, suggesting that TRF’s protective effects may be more closely linked to metabolic and structural neuronal restoration rather than growth factor modulation.

## Figures and Tables

**Table 1:** Differential expressed metabolites (DEMs) in plasma samples in non-DM control and DM rats with different treatments at 12 weeks post-intervention.

Metabolite	MW	RT	HMDB ID	Mode	Fold Change				
					DMC vs. C	DMM vs. DMC	DMT vs. DMC	DMMT vs. DMC	DMMT vs. DMT
13,14-Dihydro-15-keto Prostaglandin A2	334.21	11.64	HMDB0001244	n	3.29				
3-Hydroxybutyric acid	104.05	1.65	HMDB0000011	n	16.98				
4-Hydroxy-6-methyl-2-pyrone	126.03	4.02	HMDB0341406	n	8.35				
4-Oxoproline	129.04	1.18	HMDB0304793	n			1.68		
4-Phenylbutyric acid	164.08	6.95	HMDB0000543	p	73.06				
6-Methylquinoline	143.07	3.78	HMDB0033115	p	−1.82				
Acetyl-beta-methylcholine	159.13	0.73	HMDB0015654	p	−2.47				
Acetylcholine	145.11	0.54	HMDB0000895	p	−2.14		2.09		
Arginine	174.11	0.48	HMDB0003416/HMDB0000517	p	−2.35	2.21	1.85		
Choline	103.10	0.48	HMDB0000097	p	−1.32				
Creatine	131.07	0.54	HMDB0000064	p	−3.73				
Cytosine	111.04	0.89	HMDB0000630	p	−1.56		1.60	1.56	
Dodecanedioic acid	230.15	8.77	HMDB0000623	n	22.87				
Glycocholic acid	465.31	7.61	HMDB0000138	n	42.94				
7-Methylguanine/1-Methylguanine	165.06	1.15	HMDB0000897/HMDB0003282	p	1.48		−1.40		
Isomer: Tauroursodeoxycholic acid/Taurochenodeoxycholic acid/Taurodeoxycholic acid	499.30	9.76	HMDB0000874/HMDB0000951/HMDB0000896	n	38.48		−38.83		
Metformin	129.10	0.60	HMDB0001921	p		6.10		13.87	39.05
N-Acetyl-tryptophan	246.10	5.93	HMDB0255052	n	−2.06				
Phenylalanine	165.08	3.15	HMDB0000159	n	−1.40			1.29	
Stearic acid	284.27	11.79	HMDB0000827	n	14.91				
Tetradecanedioic acid	258.19	10.20	HMDB0000872	n	5.12				
Tryptophan	204.09	3.783	HMDB0000929/HMDB0013609/HMDB0030396	p	−1.85				

Abbreviation: C: non-DM control; DMC: DM untreated control; DMM: DM metformin; DMMT: DM metformin tocotrienol-rich fraction; DMT: DM tocotrienol-rich fraction; HMDB ID: Human Metabolome Database identification; MW: molecular weight in Dalton; n: negative ionisation mode; p: positive ionisation mode; RT: retention time in min; vs.: versus nominator as control.

**Table 2:** The pathway analysis of significant metabolites identified in this study in the (**A**) comparison between baseline and post-intervention for each group, (**B**) comparison between all group at 12 week, (**C**) comparison between DMC and control, and (**D**) comparison between DMT and DMC. Data includes the affected pathways, impact values, and *p* values, where pathways with impact values > 0.1 and *p* values < 0.05 indicate a stronger association with the observed metabolic alterations.

**(A) Comparison Baseline and Post-Intervention for All Group**			
**Pathway Name**	**Match Status**	***p* Value**	**Impact Value**
Phenylalanine, tyrosine and tryptophan biosynthesis	1/4	0.103	0.50 #
Arginine biosynthesis	4/14	0.001 *	0.30 #
Arachidonic acid metabolism	1/44	0.702	0.29 #
Tryptophan metabolism	4/41	0.022 *	0.25 #
Histidine metabolism	2/16	0.066	0.22 #
Purine metabolism	4/71	0.118	0.16 #
Tyrosine metabolism	1/42	0.685	0.14 #
Arginine and proline metabolism	2/36	0.250	0.12 #
Pyrimidine metabolism	3/39	0.084	0.11 #
Cysteine and methionine metabolism	1/33	0.595	0.10 #
Citrate cycle (TCA cycle)	2/20	0.098	0.10 #
Sphingolipid metabolism	2/32	0.210	0.08
Glycine, serine and threonine metabolism	3/34	0.060	0.07
Pentose phosphate pathway	1/22	0.452	0.05
Alanine, aspartate and glutamate metabolism	1/28	0.535	0.05
Primary bile acid biosynthesis	2/46	0.351	0.05
Glutathione metabolism	2/28	0.171	0.03
Glycerophospholipid metabolism	2/36	0.250	0.03
Steroid hormone biosynthesis	1/80	0.892	0.01
Pantothenate and CoA biosynthesis	2/20	0.098	0.01
Biosynthesis of unsaturated fatty acids	2/8	0.014	0.00
Valine, leucine and isoleucine biosynthesis	4/36	0.018	0.00
Butanoate metabolism	2/15	0.059	0.00
Taurine and hypotaurine metabolism	1/8	0.195	0.00
Phenylalanine metabolism	1/10	0.238	0.00
D-Amino acid metabolism	1/15	0.336	0.00
Ubiquinone and other terpenoid–quinone biosynthesis	1/18	0.388	0.00
beta-Alanine metabolism	1/21	0.436	0.00
Lipoic acid metabolism	1/28	0.535	0.00
Glyoxylate and dicarboxylate metabolism	1/32	0.584	0.00
Valine, leucine and isoleucine degradation	1/40	0.667	0.00
**(B) Comparison All Groups at 12 Weeks**			
**Pathway Name**	**Match Status**	***p* Value**	**Impact Value**
Phenylalanine, tyrosine and tryptophan biosynthesis	1/4	0.034 *	0.50 #
Phenylalanine metabolism	1/10	0.082	0.36 #
Arginine and proline metabolism	4/36	0.001 *	0.17 #
Tryptophan metabolism	1/41	0.298	0.14 #
Arginine biosynthesis	1/14	0.113	0.08
Sphingolipid metabolism	1/32	0.241	0.06
Glycerophospholipid metabolism	2/36	0.036 *	0.03
Primary bile acid biosynthesis	1/46	0.328	0.02
D-Amino acid metabolism	2/15	0.007 *	0.00
Glycine, serine and threonine metabolism	2/34	0.032 *	0.00
Butanoate metabolism	1/15	0.121	0.00
Biosynthesis of unsaturated fatty acids	1/36	0.267	0.00
**(C) Comparison Between DMC and Non-DM Control**			
**Pathway Name**	**Match Status**	***p* Value**	**Impact Value**
Tryptophan metabolism	1/41	0.019 *	0.14 #
Arginine and proline metabolism	1/36	0.027 *	0.12 #
Arginine biosynthesis	1/14	0.070	0.08
Glycerophospholipid metabolism	2/36	0.013 *	0.03
Primary bile acid biosynthesis	1/46	0.215	0.02
D-Amino acid metabolism	1/15	0.075	0.00
Butanoate metabolism	1/15	0.075	0.00
One carbon pool by folate	1/26	0.127	0.00
Glycine, serine and threonine metabolism	1/34	0.163	0.00
Biosynthesis of unsaturated fatty acids	1/36	0.172	0.00
**(D) Comparison Between DMT and DMC**			
**Pathway Name**	**Match Status**	***p* Value**	**Impact Value**
Arginine and proline metabolism	1/36	0.050 *	0.12 #
Arginine biosynthesis	1/14	0.036 *	0.08
Primary bile acid biosynthesis	1/46	0.114	0.02
D-Amino acid metabolism	1/15	0.038 *	0.00
Glycerophospholipid metabolism	1/36	0.090	0.00

* indicate *p* value < 0.05 and # indicate impact value > 0.1.

## Data Availability

The original contributions presented in this study are included in the article/Supplementary Materials. Further inquiries can be directed to the corresponding author.

## Supplementary Materials

The following supporting information can be downloaded at https://www.mdpi.com/article/10.3390/ijms262311247/s1.

## Abbreviations

The following abbreviations are used in this manuscript:

ACh	Acetylcholine
ACN	Acetonitrile
AGEs	Advanced Glycation End Products
ANOVA	Analysis of Variance
CDP-choline	Cytidine Diphosphate Choline
CSF	Cerebrospinal Fluid
DEM(s)	Differentially Expressed Metabolite(s)
DM	Diabetes Mellitus
DMC	Diabetes Mellitus Untreated Control
DMM	Diabetes Mellitus + Metformin
DMT	Diabetes Mellitus + Tocotrienol-Rich Fraction
DMMT	Diabetes Mellitus + Metformin + Tocotrienol-Rich Fraction
DPN	Diabetic Peripheral Neuropathy
EDTA	Ethylenediaminetetraacetic Acid
ELISA	Enzyme-Linked Immunosorbent Assay
ER	Endoplasmic Reticulum
FA	Fatty Acids
HFD	High-Fat Diet
HODE	Hydroxyoctadecadienoic Acid
IENFD	Intraepidermal Nerve Fibre Density
IP	Intraperitoneal
IVC	Individually Ventilated Cage
KEGG	Kyoto Encyclopaedia of Genes and Genomes
KTX	Ketamine–Xylazine–Tiletamine–Zolazepam
LARU	Laboratory Animal Resource Unit
LC-MS/MS	Liquid Chromatography Tandem Mass Spectrometry
MASLD	Metabolic Dysfunction-Associated Steatotic Liver Disease
MF(s)	Metabolite Feature(s)
MS	Mass Spectrometry
m/z	Mass-to-Charge Ratio
MW	Molecular Weight
NAD+	Nicotinamide Adenine Dinucleotide
NEFL	Neurofilament Light Chain
NGF	Nerve Growth Factor
PCA	Principal Component Analysis
PGE_2_	Prostaglandin E_2_
PGI_2_	Prostaglandin I_2_
PG	Prostaglandin
PPARγ	Peroxisome Proliferator-Activated Receptor Gamma
QC	Quality Control
ROS	Reactive Oxygen Species
RT	Retention Time
SD	Sprague Dawley
SFN	Small Fibre Neuropathy
STZ	Streptozotocin
TCA	Tricarboxylic Acid
TCDCA	Taurochenodeoxycholic Acid
TGR5	Takeda G-Protein-Coupled Receptor 5
T2DM	Type 2 Diabetes Mellitus
TRF	Tocotrienol-Rich Fraction
UHPLC-MS/MS	Ultra-High-Performance Liquid Chromatography Tandem Mass Spectrometry
UKMAEC	Universiti Kebangsaan Malaysia Animal Ethics Committee
UPLC/QTOF-MS	Ultra-Performance Liquid Chromatography–Quadrupole Time-of-Flight Mass Spectrometry
