# Supplementary material for: Exploring the Effects of Palm Tocotrienol-Rich Fraction in Diabetic Peripheral Neuropathy Rat’s Model: An Untargeted Metabolomic Profiling and Correlation Study
Source: Int J Mol Sci. 2025 Nov 21;26(23):11247. doi: 10.3390/ijms262311247 (PMC12692528; doi:10.3390/ijms262311247)
Supplement: Supplementary file 1 [file ijms-26-11247-s001.zip › ijms-3898343-supplementary.pdf]

Table S1: Changes in body weight of the non-DM control and DM groups with different treatment interventions from 0 (baseline) to 12-week post-intervention.

Week	Body weight (g)				
	Control	DMC	DMM	DMT	DMMT
0	399.08±44.43	324.00±51.63	323.13±48.84	317.50±39.01	312.50±29.64
1	408.75±47.06	322.50±56.57	329.38±47.54	316.88±38.63	305.63±28.34
2	417.08±44.44	331.25±61.16	335.00±51.69	333.75±37.77	321.25±34.62
3	424.58±44.85	335.00±52.28	345.63±51.99	345.63±46.71	328.75±44.62
4	435.00±46.51	332.14±47.68	352.50±66.22	350.00±45.28	331.25±46.66
5	445.00±53.47	337.14±47.33	352.50±70.96	358.75±44.14	337.50±52.64
6	448.17±56.40	338.57±45.43	371.43±69.51	353.13±54.77	334.38±56.22
7	457.08±55.66	337.14±45.54	359.29±77.91	355.00±53.12	338.13±54.70
8	465.00±58.15	340.00±44.72	359.29±69.31	362.50±54.90	337.50±56.63
9	465.83±58.15	315.71±54.65	352.14±71.93	362.50±57.20	336.88±52.78
10	467.50±59.98	327.86±48.81	357.86±74.32	364.38±60.44	335.63±51.99
11	468.33±65.66	305.71±54.73	355.71±82.33	359.38±69.87	336.25±56.24
12	468.33±66.99	297.14±58.73	346.43±90.95	354.38±72.77	327.50±57.45

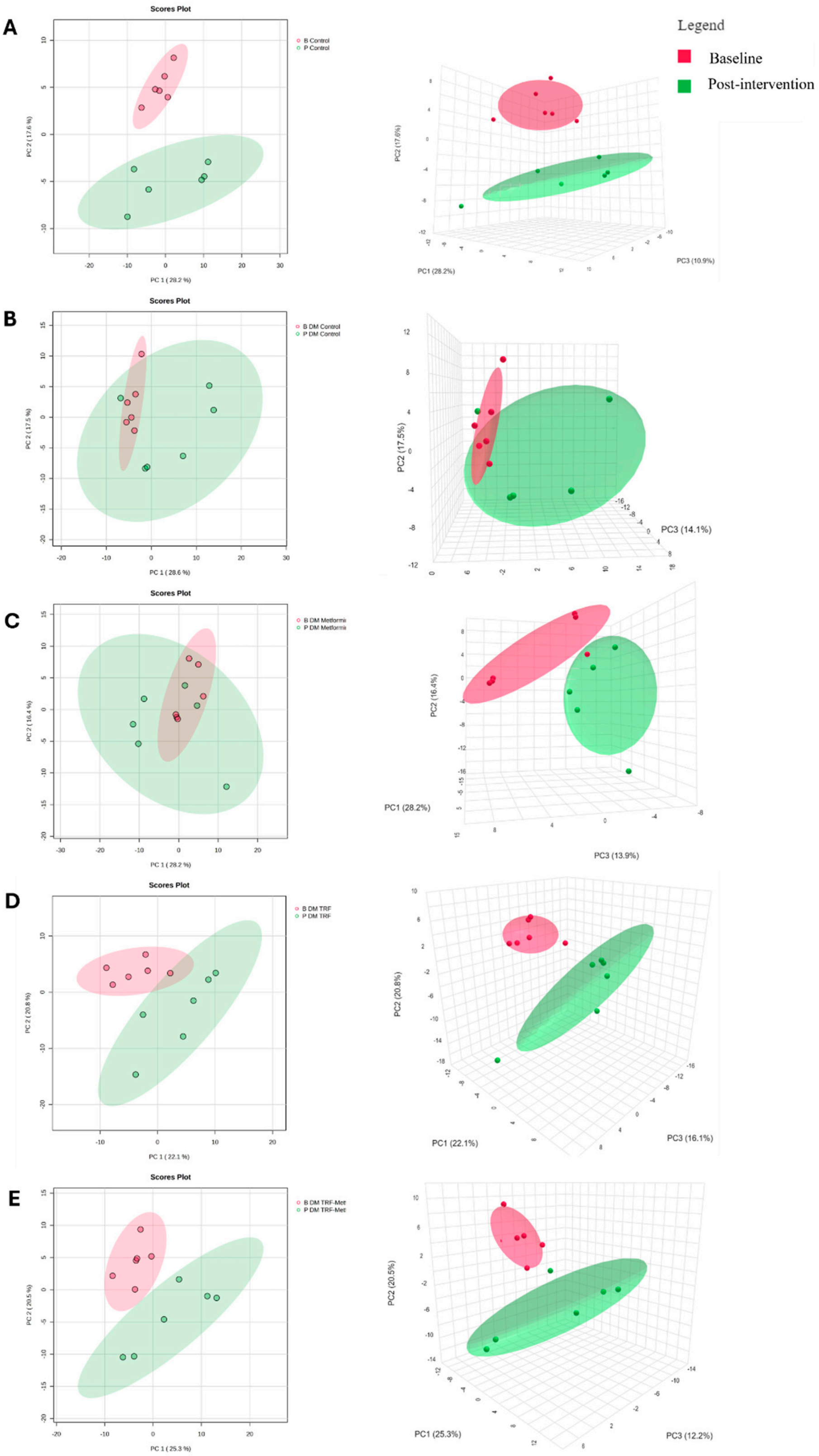

Figure S1: The score plots of PCA (plasma) on different groups between baseline and post-treatment where A) Non-DM control, B) DM untreated Control C) DM Metformin, D) DM TRF, E) DM TRF-Metformin, each with 2D and 3D respectively, *n*=6 per group.

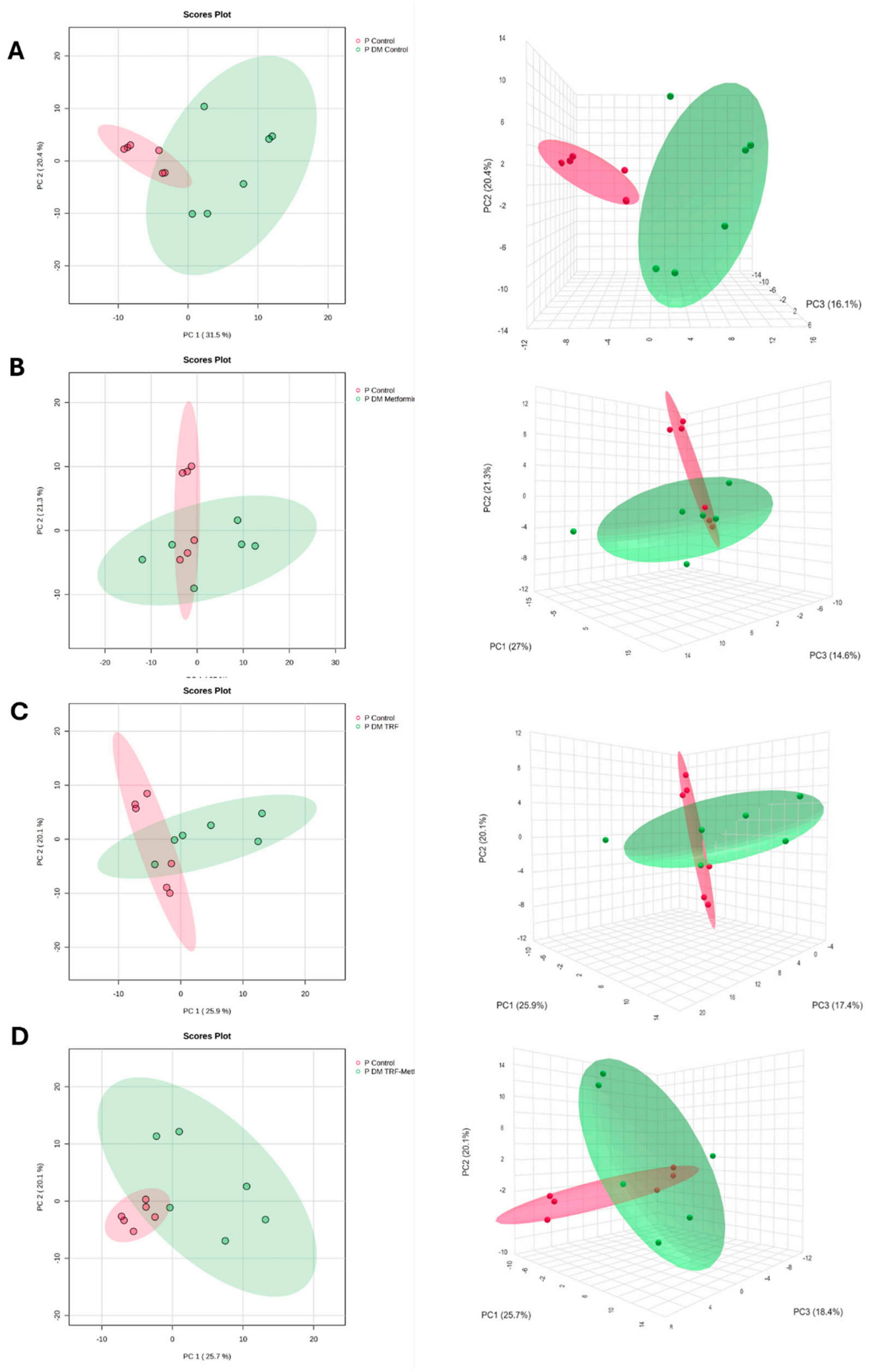

Figure S2: The PCA score plots of plasma samples at post-treatment between non-DM control and (A) DM untreated control, (B) DM Metformin, (C) DM TRF and (D) DM TRF-Metformin, with *n*=6 per group

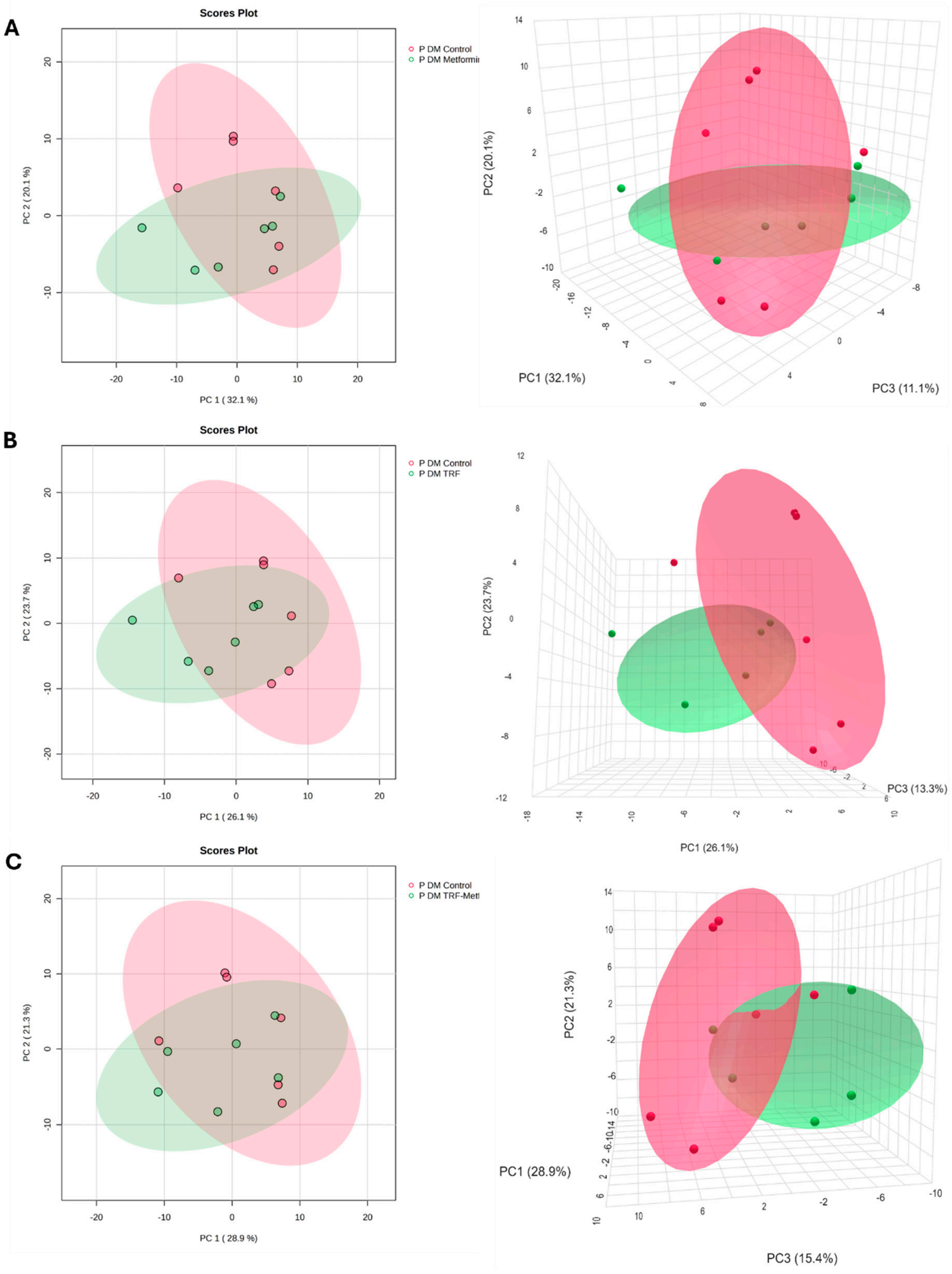

Figure S3: The score plots of PCA (plasma) at post-treatment between treatment groups and DM untreated control where A) DM Met B) DM TRF and C) DM TRF-Met respectively, n=6 per group.

Table S2: Differential express metabolites (DEMs) in plasma samples in rats with DPN between baseline and post-intervention in each group. Analysis was performed using one-way ANOVA with Turkey’s post hoc analysis.

Metabolite	MW	RT	HMDB ID	mode	C	DMC	DMM	DMT	DMMT
(+/-)13-HODE	296.24	12.48	HMDB0004667	n	-1.54		2.81	2.98	
(+/-)9,10-dihydroxy-12Z-octadecenoic acid	314.25	11.17	HMDB0004704	n	-2.24		2.94		
13,14-Dihydro-15-keto Prostaglandin A2	334.21	11.64	HMDB0001244	n					-1.53
16-Hydroxyhexadecanoic acid	272.24	14.70	HMDB0006294	n		-1.18	-1.49		
2-Oxoglutaric acid	146.02	0.82	HMDB0000208	n		-2.49			
2-Hydroxy-4-methylthiobutanoic acid	150.03	3.85	HMDB0037115	n				15.21	
3-Hydroxybutyric acid	104.05	1.65	HMDB0000011	n	3.22				
3-Indoxyl sulphate	213.01	5.10	HMDB0000682	n			-1.51		
4',7-Dihydroxyflavanone	256.07	5.66	HMDB0029519	p			6.55	18.96	
4-Methylphenol	108.06	5.74	HMDB0001858	n			-1.90		
4-Oxoproline	129.04	1.18	HMDB0304793	n				1.51	
4-Phenolsulfonic acid	173.99	4.25	HMDB0304953	n					7.53
4-Phenylbutyric acid	164.08	6.95	HMDB0000543	p				-7.91	-3.60
4-guanidinobutyric acid	145.09	0.73	HMDB0003464	p	-1.79				
5-Hydroxyindole-3-acetic acid	191.06	2.98	HMDB0000763	p				-1.53	
5-Methycytosine	125.06	1.41	HMDB0002894	p					1.51
6-Hydroxycaproic acid	132.08	5.09	HMDB0012843	n				1.91	2.04
6-Methylquinoline	143.07	3.78	HMDB0033115	p		-1.35		-1.25	
6-Methylnicotinamide	136.06	0.50	HMDB0013704	p					3.09

Table S2: Continue.

Metabolite	MW	RT	HMDB ID	mode	C	DMC	DMM	DMT	DMMT
Acetylcholine	145.11	0.538	HMDB0000895	p	1.36				
Arachidonic acid	304.24	15.269	HMDB0001043	p		1.47			
Arginine	174.11	0.477	HMDB0003416/ HMDB0000517	p	-1.19	-2.53			
Acetyl-carnitine	203.12	0.855	HMDB0000201/ HMDB0240771	p					1.42
Ascorbic acid 2-sulfate	255.99	0.789	HMDB0060649	n			-1.55		
Betaine	117.08	0.671	HMDB0000043	p		2.58		2.07	3.43
Catechol	110.04	3.804	HMDB0000957	n			9.69	9.26	10.11
Choline	103.10	0.484	HMDB0000097	p	1.19				1.55
Citrulline	175.10	0.504	HMDB0000904	p	-1.21				
Cytidine	243.09	0.74	HMDB0000089	p	1.95				2.34
Creatinine	113.06	0.51	HMDB0000562	p				-1.23	
Corticosterone	346.21	8.64	HMDB0001547	p		1.43			
Coumarin	146.04	1.35	HMDB0001218	p		-1.36			
Cyclohexanecarboxylic acid	128.08	5.90	HMDB0031342	n		3.21			
Decanoylcarnitine	315.24	8.03	HMDB0000651	p	1.72				1.83
Docosahexaenoic acid	328.24	15.07	HMDB0002183	p		2.05	1.98		1.39
Dodecanedioic acid	230.15	8.77	HMDB0000623	n					-1.98
Eicosapentaenoic acid	302.22	12.96	HMDB0001999	p		3.78			
Erythro-sphingosine 1-phosphate	379.25	10.99	HMDB0000277	p				1.52	

Table S2: Continue.

Metabolite	MW	RT	HMDB ID	mode	C	DMC	DMM	DMT	DMMT
Ethyl-beta-glucuronide	222.07	1.610	HMDB0010325	n					2.78
Guanidinosuccinic acid	175.06	1.66	HMDB0003157	p				1.33	
Glutathione oxidized	612.15	1.58	HMDB0003337	n		-2.16			
Hexanoylcarnitine	259.18	5.29	HMDB0000756	p				1.50	1.36
Hippuric acid	179.06	4.71	HMDB0000714	n		5.78	6.78	4.68	2.14
Hypoxanthine	136.04	0.98	HMDB0000157	p				2.79	
Hexadecanamide	255.26	15.06	HMDB0012273	p	1.32				
Histidine	155.07	0.47	HMDB0000177	n	-1.34				
Indole	117.06	4.71	HMDB0000738	p		7.31	7.58	5.08	7.27
Indole-3-acetic acid	175.06	6.49	HMDB0000197	p				-1.65	
Indole-3-acrylic acid	187.06	7.03	HMDB0000734	n		2.76		2.22	
Indole-3-lactic acid	205.07	5.86	HMDB0000671	n				1.20	
Isocitric acid	192.03	0.66	HMDB0000193	n	-1.65				
Inosine-5'-monophosphate	348.05	0.99	HMDB0000175	n				3.59	
Isomer: 1-Methylhistidine/alpha-Methyl-histidine	169.09	0.49	HMDB0000001	p				-1.61	-1.81
Isomer: 3,5-Dihydroxybenzoic acid/2,4-Dihydroxybenzoic acid	154.03	5.10	HMDB0013677/ HMDB0029666	n		5.96			4.44
Isomer: 3-Coumaric acid/4-Coumaric acid/2-Hydroxycinnamic acid	164.05	5.35	HMDB0001713/ HMDB0002035/ HMDB0002641	n			2.46		3.43
Isomer: Ferulic acid/Isoferulic acid	194.06	4.78	HMDB0000954/ HMDB0000955	n	-2.28			7.99	8.57

Table S2: Continue.

Metabolite	MW	RT	HMDB ID	mode	C	DMC	DMM	DMT	DMMT
Isomer: Tauroursodeoxycholic acid/Taurochenodeoxycholic acid/Taurodeoxycholic acid	499.30	9.76	HMDB0000874/ HMDB0000951/ HMDB0000896	n				-4.33	
Isomer: Gluconic acid/Galactonic acid	196.06	0.51	HMDB0000625/ HMDB0000565	n		2.17			
Isoquinoline	129.06	7.35	HMDB0034244	p		3.09		3.08	
Kynurenine	208.09	2.98	HMDB0000684/ HMDB0000684	p				-1.53	
Metformin	129.10	0.60	HMDB0001921	p			6.71		23.41
Methionine	149.05	0.96	HMDB0000696	p	-1.64			-1.64	-0.61
Methyl indole-3-acetate	189.08	7.35	HMDB0029738	p		3.09		3.05	
N-Acetyl-carnosine	268.12	0.83	HMDB0012881	p		-2.84	-4.51	-2.55	-2.71
N-Acetylornithine	174.10	0.62	HMDB0003357	p	-1.77		4.76	4.25	
N-Acetyl-tryptophan	246.10	5.93	HMDB0255052	n					1.417
Pantothenic acid	219.11	3.59	HMDB0000210	n			1.85	3.61	
Phenylalanine	165.08	3.15	HMDB0000159	n				-1.18	
Phenyllactic acid	166.06	5.64	HMDB0000563	n		2.57		2.22	3.82
Propionylcarnitine	217.13	1.97	HMDB0000824	p	-1.87			1.52	
Pyroglutamic acid	129.04	1.21	HMDB0000267	p	1.33			1.79	1.66
Sphingosine	299.28	9.91	HMDB0000252	p			1.918	1.655	2.05

Table S2: Continue.

Metabolite	MW	RT	HMDB ID	mode	C	DMC	DMM	DMT	DMMT
Suberic acid	174.09	5.638	HMDB0000893	n		-4.26	-4.64		
Stearic acid	284.27	11.79	HMDB0000827	n				2.77	
Taurocholic acid	515.29	7.90	HMDB0000036	p	-2.10				
Tetradecanedioic acid	258.18	10.20	HMDB0000872	n					-1.63
Threonine	119.06	0.50	HMDB0000167	p		-1.51		-1.37	-1.35
Thymidine	242.09	3.34	HMDB0000273	n		-1.53			
Tryptophan	204.09	3.78	HMDB0000929/ HMDB0013609/ HMDB0030396	p		-1.41	-1.41	-1.32	
Tyrosine	181.07	1.35	HMDB0000158	p		-1.35			
Uric acid	168.03	0.98	HMDB0000289	p	2.46				2.78
Uridine	244.07	1.52	HMDB0000296	n	3.08				4.36
Valine	117.08	0.76	HMDB0000883	p					4.66
Xanthosine	284.08	3.10	HMDB0000299	n	2.83	1.86			

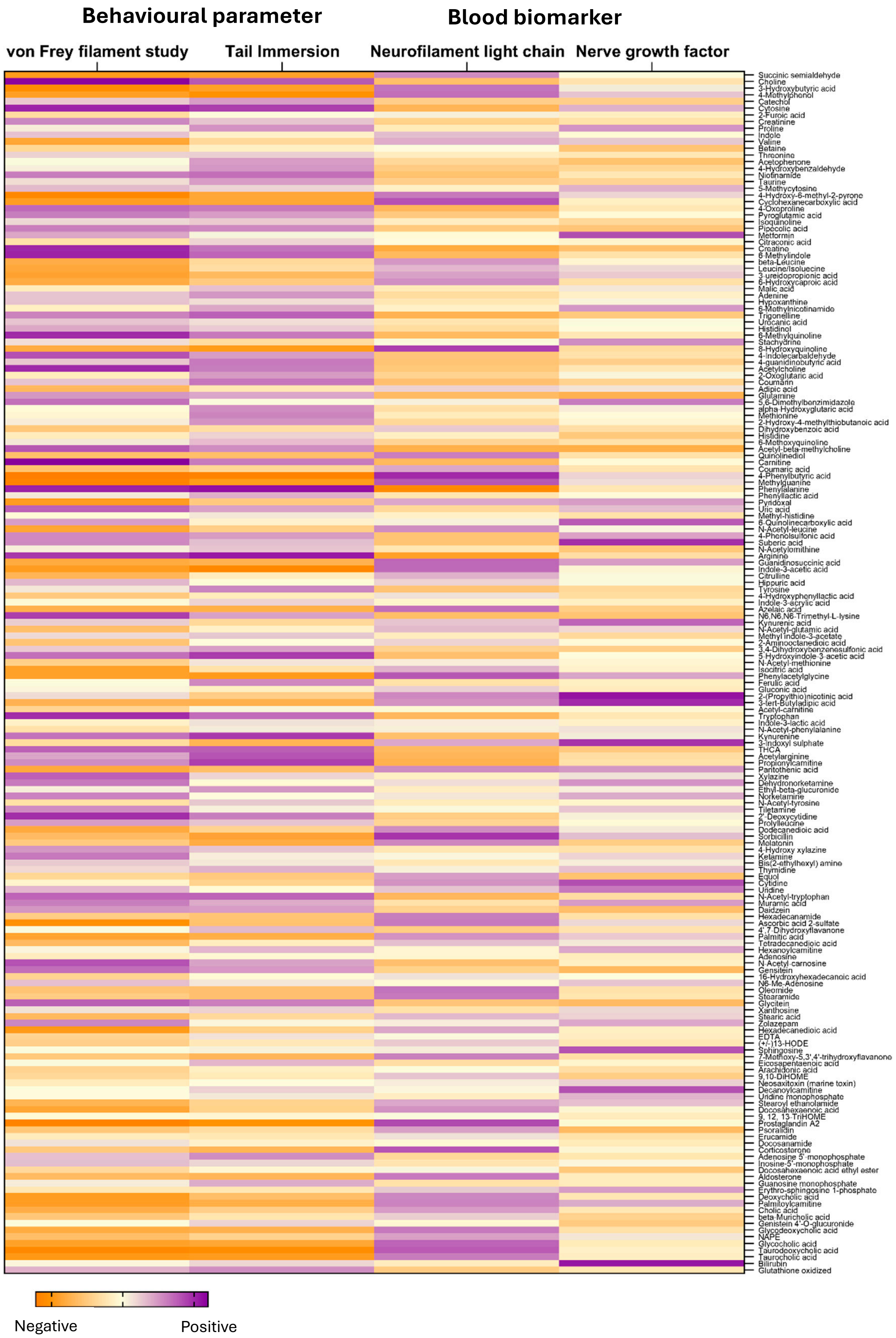

Figure S4: Correlation analysis between 180 metabolite levels with behavioral and blood parameters in DPN, illustrated in a heatmap.

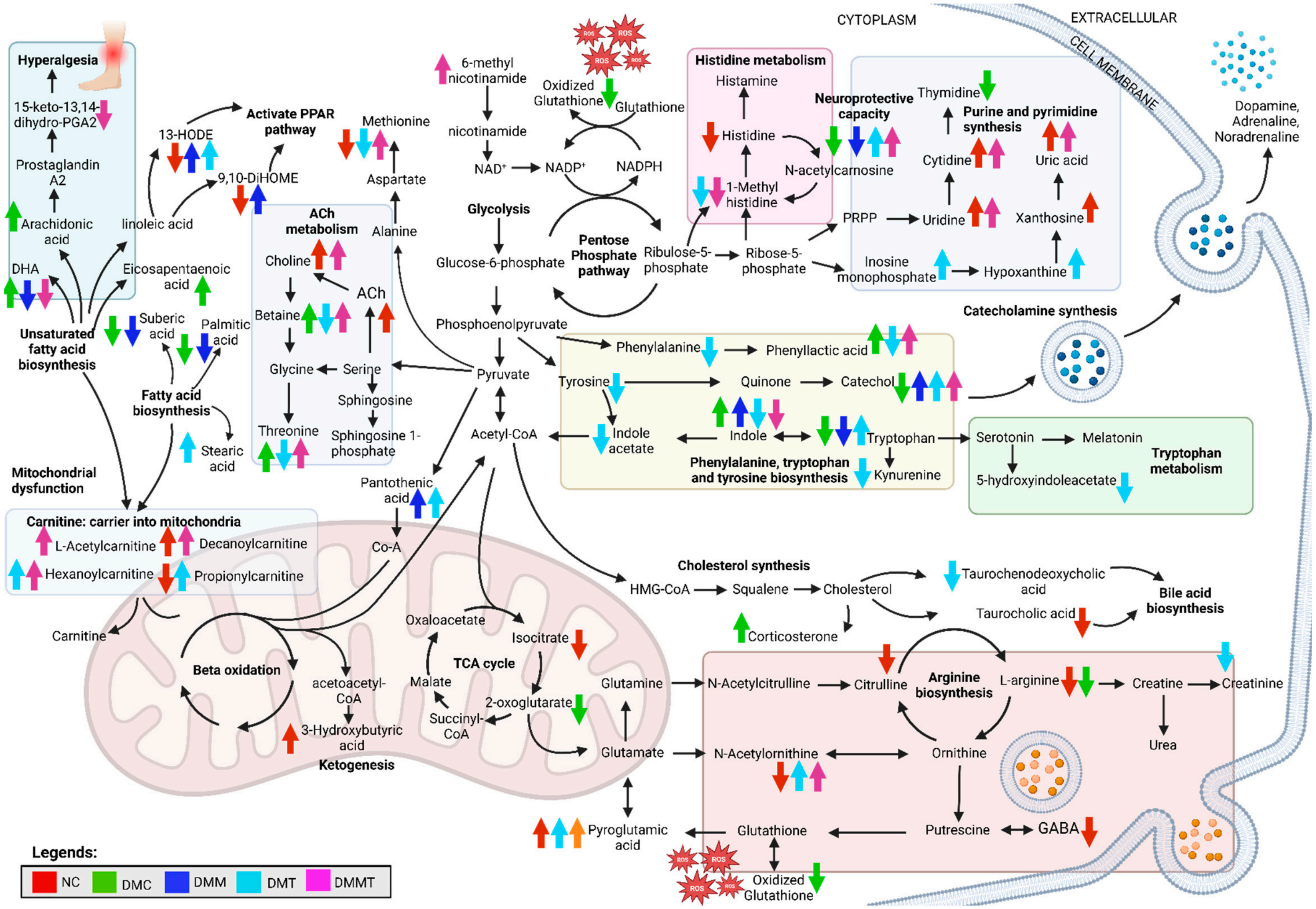

Figure S5: Summary of significant differential express metabolites (DEMs) in DPN animal model between baseline and post-intervention. Analysis was performed using one-way ANOVA with Turkey's post hoc analysis, where n=6 per group. Created with Biorender.com, accessed on 12 September 2025.

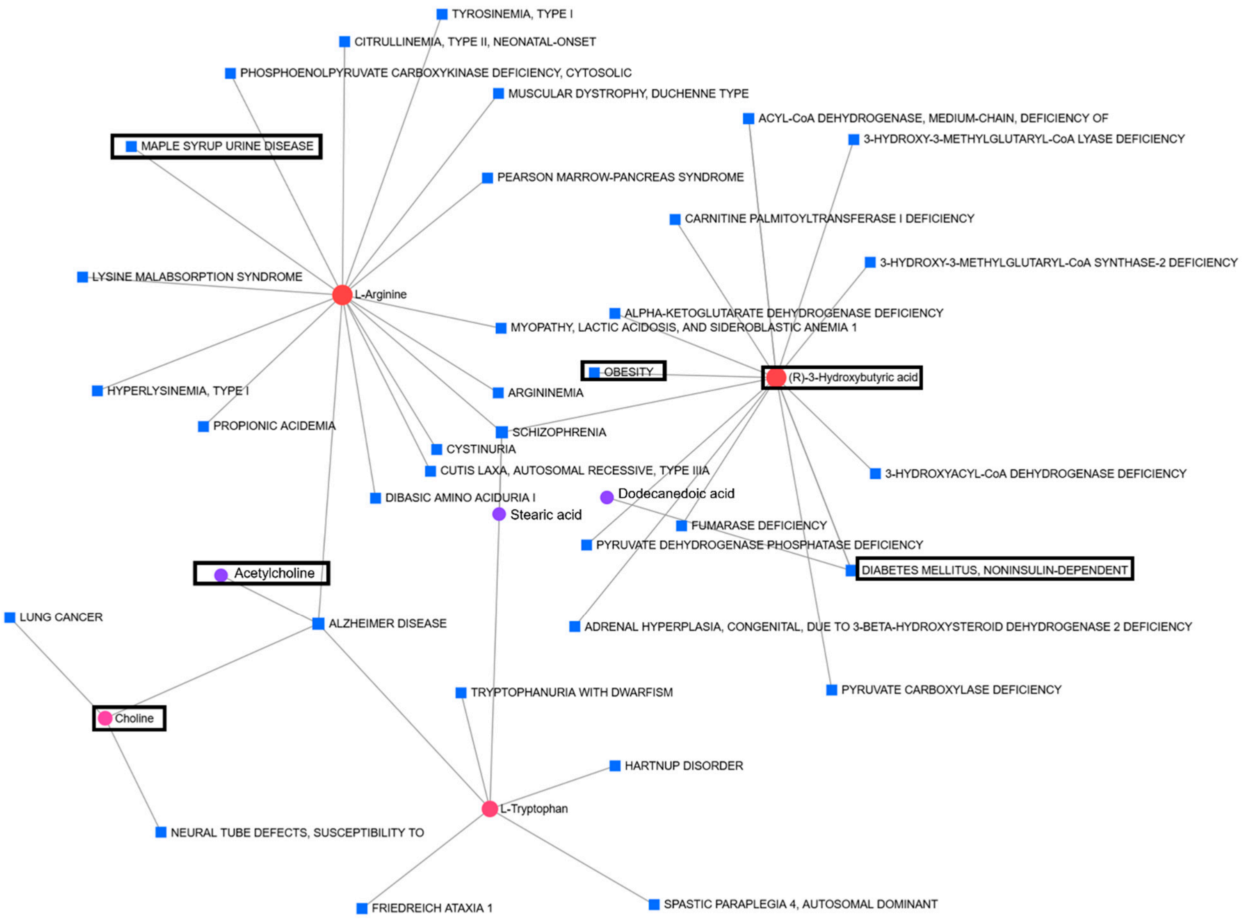

Figure S6: Network analysis on the metabolites-disease network

Table S3: Differential express metabolites (DEMs) in plasma samples in non-DM control and DM rats with different treatments at 12 weeks post-intervention with respective p value and FDR where * $p < 0.05$ and # $q < 0.05$.

Metabolite	p value	FDR
13,14-Dihydro-15-keto Prostaglandin A2	0.029*	0.200
3-Hydroxybutyric acid	0.031*	0.200
4-Hydroxy-6-methyl-2-pyrone	0.023*	0.192
4-Oxoproline	0.031*	0.200
4-Phenylbutyric acid	0.000*	0.003#
6-Methylindole	0.007*	0.095
6-Methylquinoline	0.004*	0.095
Acetyl-beta-methylcholine	0.014*	0.130
Acetylcholine	0.013*	0.128
Arginine	0.006*	0.095
Choline	0.049*	0.257
Creatine	0.005*	0.095
Cytosine	0.012*	0.127
Dodecanedioic acid	0.000*	0.002#
Glycocholic acid	0.028*	0.199
Isomer: 7-Methylguanine/1-Methylguanine	0.008*	0.095
Isomer: Tauroursodeoxycholic acid/Taurochenodeoxycholic acid/Taurodeoxycholic acid	0.000*	0.008#
Metformin	0.000*	0.001#
N-Acetyl-tryptophan	0.003*	0.085
Phenylalanine	0.003*	0.085
Stearic acid	0.008*	0.095
Tetradecanedioic acid	0.003*	0.085
Tryptophan	0.007*	0.095
